# Co-carriage of *Staphylococcus aureus*, *Streptococcus pneumoniae*, *Haemophilus influenzae* and *Moraxella catarrhalis* among three different age categories of children in Hungary

**DOI:** 10.1371/journal.pone.0229021

**Published:** 2020-02-07

**Authors:** Eszter Kovács, Judit Sahin-Tóth, Adrienn Tóthpál, Mark van der Linden, Tamás Tirczka, Orsolya Dobay

**Affiliations:** 1 Institute of Medical Microbiology, Semmelweis University, Budapest, Hungary; 2 German National Reference Center for Streptococci, Department of Medical Microbiology, University Hospital RWTH Aachen, Aachen, Germany; 3 National Public Health Center, Budapest, Hungary; Universidade de Lisboa Faculdade de Medicina, PORTUGAL

## Abstract

**Background:**

The nasopharynx can from time to time accommodate otherwise pathogenic bacteria. This phenomenon is called asymptomatic carriage. However, in case of decreased immunity, viral infection or any other enhancing factors, severe disease can develop. Our aim in this study was to survey the nasal carriage rates of four important respiratory pathogens in three different age groups of children attending nurseries, day-care centres and primary schools. This is the first study from Hungary about the asymptomatic carriage of *H*. *influenzae* and *M*. *catarrhalis*.

**Methods:**

Altogether 580 asymptomatic children were screened in three Hungarian cities. Samples were collected from both nostrils with cotton swabs. The identification was based on both colony morphology and species-specific PCRs. Serotyping was performed for *S*. *pneumoniae*, *H*. *influenzae* and *M*. *catarrhalis*. Antibiotic susceptibility was determined with agar dilution, according to the EUCAST guidelines. Clonality was examined by PFGE.

**Results and conclusions:**

Whereas the carriage rates of *S*. *pneumoniae*, *H*. *influenzae* and *M*. *catarrhalis* clearly decreased with age, that of *S*. *aureus* showed an opposite tendency. Multiple carriage was least prevalent if *S*. *aureus* was one of the participants. The negative association between this bacterium and the others was statistically significant. For pneumococcus, the overall carriage rate was lower compared to earlier years, and PCV13 serotypes were present in only 6.2% of the children. The majority of *H*. *influenzae* isolates was non-typeable and no type b was detected; serotype A was dominant among *M*. *catarrhalis*. All four bacteria were more sensitive to antibiotics compared to clinical isolates. No MRSAs were detected, but we found three mupirocin resistant strains. The positive effect of Hib- and PCV-vaccination is undoubted. Continued surveillance of these pathogens is required.

## Introduction

The human nasopharynx provides a niche for different bacteria including some potentially pathogenic species. Asymptomatic carriage of *Streptococcus pneumoniae*, *Staphylococcus aureus*, *Haemophilus influenzae* and *Moraxella catarrhalis* can precede the development of severe respiratory tract infections as well as invasive diseases [1–4]. Nasopharyngeal carriage occurs mostly in children attending communities where crowding favours the horizontal spread of these bacteria. These young children are then the primary reservoirs for infections developing in other susceptible individuals, such as grandparents [5, 6]. The infections caused by these bacteria include pneumonia, otitis media, sepsis and meningitis.

Meta-analysis of numerous randomized controlled trials and observational studies between 1970 and 2010 among children under five years of age in developing countries showed that pneumococcal conjugated vaccines (PCVs) and *Haemophilus influenzae* type b (Hib) vaccine could prevent approximately three-quarters of meningitis-related and 18–26% of pneumonia-related deaths [7, 8]. Beside the reduction of diseases caused by vaccine serotypes (VTs), a similar decrease was also observed in asymptomatic carriage. However, parallel to this, the ratio of non-vaccine serotypes has increased in both infection and carriage [9, 10].

According to the Hungarian vaccination schedule, Hib has been mandatory since 1999 in a 3+1 scheme (2, 3, 4 + 18 months) and the vaccination rate in the population has been very high (99.5–99.8%) from the beginning [11, 12]. PCVs were gradually introduced: PCV7 was launched in 2005, but it was made free of charge for children under 2 years of age only in 2008. It became a recommended vaccine in April 2009, and 1.5 years later it was replaced by PCV13. In July 2014, PCV13 was integrated in the national immunization program in a 2+1 scheme (2, 4 + 12 months) as a mandatory vaccine [13, 14]. Vaccination rates among the target population jumped quickly to >85% already in 2009 and increased further to 99.6–99.9% by 2018 [15–17].

The direct benefit of the vaccines is well understood, but it is hard to predict the future consequences of the eradication of vaccine types; which serotypes become dominant, how the dynamics and the competitive relationship between other species will change. The aim of this study was to characterize the co-colonizing *S*. *pneumoniae*, *S*. *aureus*, *H*. *influenzae* and *M*. *catarrhalis* isolates in three different age groups with different PCV vaccination status.

## Materials and methods

### Study population

Altogether 580 children were screened for the four respiratory tract pathogens. The genders were very much equalised: there were 287 females and 285 males (the gender was not provided in 8 cases). The children were divided into three age groups: n = 336 attending nurseries (1–3 years old), n = 186 attending DCCs (day-care centres (3–6 years old)) and n = 58 attending primary school (6–13 years old). Institutions of three Hungarian cities, Budapest, Székesfehérvár and Pápa, were involved in the survey between March 2015 and May 2016 (ten nurseries, ten DCCs and one primary school). These three cities represent three different population size: the capital, a chief county town and a small town. Parents were informed about the purpose of the study and they were requested to fill out a questionnaire about potential risk factors for carriage and to sign a document of permission. Questions were asked about vaccination status, gender, having siblings, antibiotic exposure in the past two weeks and passive smoking. The Fisher’s exact test was applied for statistical analysis.

### Specimen collection

For sample collection, both nostrils were wiped with the same sterile cotton swab per child. The swabs were inserted into active charcoal containing semi-solid medium (Transwab, Medical Wire & Equipment, Corsham, UK) and transported to the laboratory on the same day.

### Phenotypical identification

The samples were inoculated onto Columbia blood agar plates (for *S*. *pneumoniae* and *S*. *aureus*) and in parallel onto vancomycin containing chocolate agar plates for the selective cultivation of *H*. *influenzae* and *M*. *catarrhalis*. After an overnight incubation at 37°C in 5% CO_2_, colonies showing typical species-specific morphology were chosen to produce pure cultures. Pneumococcal-like colonies (α-haemolysis on blood agar, mucoid or flat colonies collapsed in the middle) were further tested for optochin sensitivity (5μg discs, Mast Group Ltd., Bootle, UK). β-haemolytic colonies on blood agar were identified as *S*. *aureus* by catalase and clump test (Pastorex Staph-Plus Kit, Bio-Rad, Marnes-la-Coquette, France). The smooth, round, colourless or greyish colonies of *H*. *influenzae* on chocolate agar plates supplemented with both X- and V-factors were confirmed with a positive catalase and oxidase test. Oxidase test was used also for the identification of *M*. *catarrhalis*, which often grew as pure culture on vancomycin containing chocolate agar showing the “hockey puck sign” (i.e., sliding along the surface without hindrance). The phenotypically confirmed isolates were frozen and stored at -80°C on cryobeads (Cryobank, Mast Group Ltd., Bootle, UK) until further testing.

### Genotypical identification

Species-specific PCR was applied for the genotypical identification of each bacterium. The following gene targets were used: *nucA* for *S*. *aureus*, *lytA* for *S*. *pneumoniae*, *ompP2* for *H*. *influenzae* and *16S rRNA* for *M*. *catarrhalis* [18–21]. *MecA* was simultaneously detected with *nucA* in an in-house duplex PCR to screen for methicillin-resistant *S*. *aureus* isolates [18].

### Serotyping

Pneumococcus serotyping was done primarily with the Pneumotest-Latex kit (Statens Serum Institut, Copenhagen, Denmark), complemented with PCR using primers described by the CDC or others [22, 23]. Difficult strains were sent to the German National Reference Centre for Streptococci (GNRCS), Aachen, or the National Public Health Center, Budapest. The ability of capsule expression of *H*. *influenzae* isolates was checked by the PCR detection of *bexA* gene, and in case of positivity, the isolates were further tested to differentiate capsular types (a-f) as reported by Falla et al. [24]. PCR based serotype determination was also used for *M*. *catarrhalis* isolates. For serotype A, the primers by Edwards et al. were applied [25]. In case of serotypes B and C, we made some modifications and a new primer pair was designed with the Primer3 program (forward: 5’-ACTGCCTGTGGCTTTATGCT-3’ and reverse: 5’-TCGAAGACGCACTTTAGCTG-3’), which resulted in different size of amplicons: 1488 bp for serotype B and 2485 bp for serotype C.

### Antibiotic susceptibility testing

The MIC of the isolates was determined by the agar dilution method using an A400 multipoint inoculator (AQS Manufacturing Ltd., Southwater, UK) on Mueller-Hinton agar plates (for *S*. *aureus*) or horse blood agar plates supplemented with NAD (for the other three species). For each species, the appropriate incubation circumstances and control strains were applied. The results were interpreted according the EUCAST guidelines [26]. The following antibiotics were tested, where appropriate: penicillin, ampicillin, amoxicillin-clavulanic acid, oxacillin, cefotaxime, imipenem, tetracycline, erythromycin, clindamycin, gentamicin, ciprofloxacin, levofloxacin, moxifloxacin, vancomycin, mupirocin and trimethoprim-sulfamethoxazole. Nitrocefin disks (Sigma-Aldrich, St. Louis, USA) were used to detect β-lactamase production of ampicillin resistant *H*. *influenzae* isolates. The *S*. *aureus* isolates with an oxacillin MIC ≥0.25 mg/L were also screened with 30 μg cefoxitin discs (Bio-Rad).

Mupirocin resistant *S*. *aureus* isolates were further tested by PCR to distinguish low-level resistance (due to point mutations in the chromosomal isoleucyl-tRNA synthetase (*ileS*) gene causing amino acid changes) and high-level resistance (due to presence of a plasmid containing a gene, called *mupA* or *ileS2*) [27, 28]. The reverse primer for *ileS* was changed to 5’–AAGATTGGTGCTAACAACTTCGTCATA -3’ to amplify a 790 bp fragment. The *ileS* PCR products were purified by the QIAquick PCR purification kit (Qiagen, Germany) and sent for sequencing to BIOMI Ltd., Gödöllő, Hungary. The sequences were compared to that of a mupirocin sensitive reference strain, ATCC 25923 (GenBank accession no. CP009361.1) using BLAST.

### Genotyping

All *S*. *aureus* isolates were genotyped by pulsed-field gel electrophoresis (PFGE) using the *Sma*I digestion enzyme (Sigma, Poole, Dorset, UK) as described before [29]. PFGE of the *H*. *influenzae* isolates was performed similarly, with some supplementation taken from the protocol by Saito et al [30]. Briefly, after solidification, the plugs were incubated first in 100 mM EDTA containing lysozyme for three hours at 37°C, then in 100 mM EDTA containing proteinase K overnight at 54°C. The purified chromosomal DNA was digested by *SmaI* enzyme. DNA fragments were separated in 1% pulse-field certified agarose gel (Bio-Rad) in 0.5x TBE (Tris-boric acid-EDTA) buffer in a CHEF-DR® II apparatus, at 14°C, 6 V/cm, for 10 h with pulse times of 5 s to 15 s, and for 10.5 h with pulse times of 15 s to 60 s. *S*. *pneumoniae* isolates were also compared based on their *SmaI* restriction patterns as described before [31]. PFGE profiles were analysed with the BioNumerics software version 2.5 (Applied Maths, Sint-Martens-Latem, Belgium), applying unweighted pair group method using arithmetic averages (UPGMA) and the different bands similarity coefficient, with a band position tolerance of 2.0%. During interpretation, the Tenover’s criteria [32] and the suggested designation by van Belkum et al. [33] were applied.

### Ethics statement

The number of the ethical permit was TUKEB 4-4/2009, issued by the Regional and Institutional Committee of Science and Research Ethics of Semmelweis University, Budapest. Only those children were enrolled in the study, whose parents provided written informed consent on the child’s behalf.

## Results

### Carriage rate

**Fig 1:**
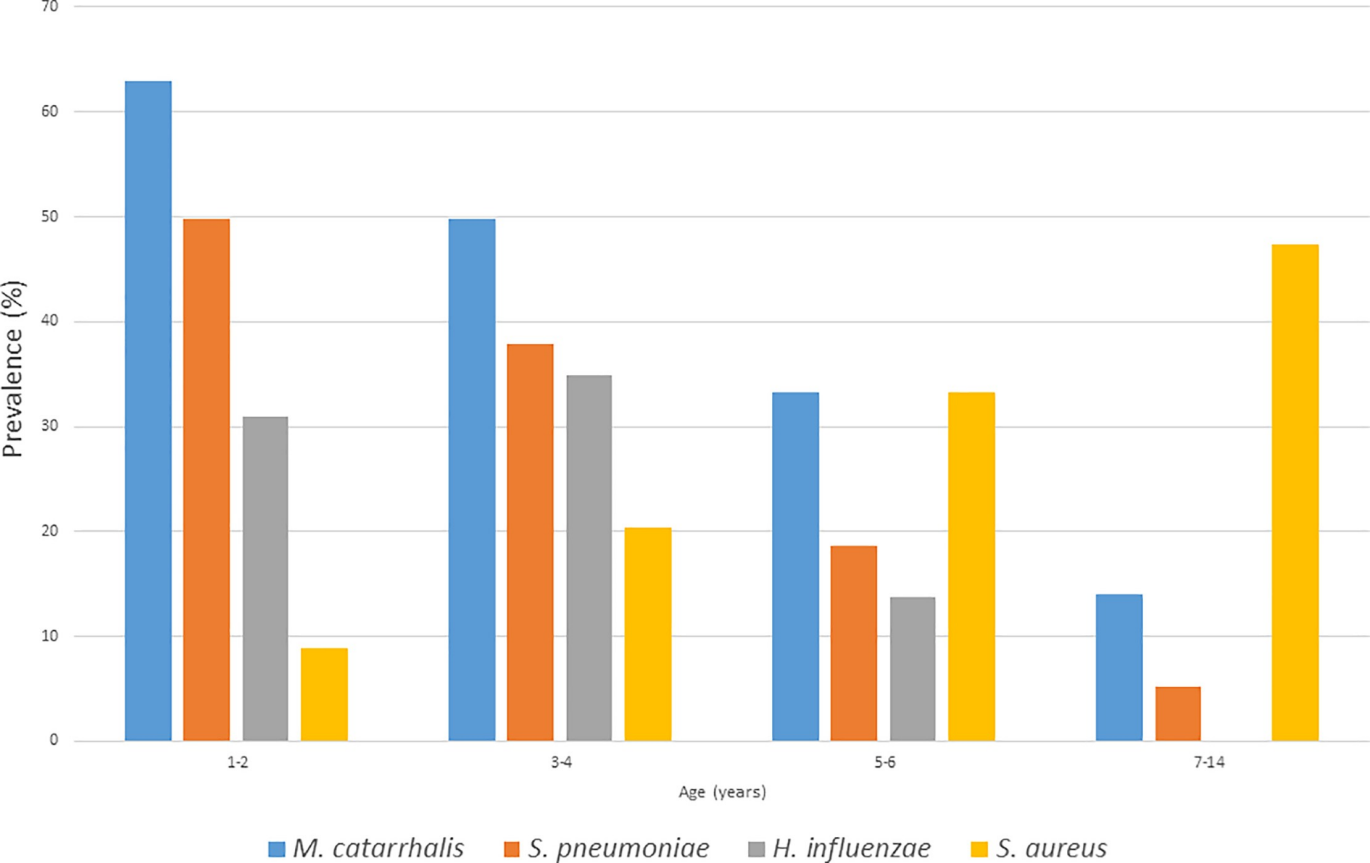
Carriage prevalence changes of the four bacterial species related to age.

Among 580 children, 442 (76.2%) carried at least one of the four bacterial species. Individual carriage rates of each bacterium showed an age-related tendency and there was a clear inverse correlation between the prevalence of *S*. *aureus* and the other three species (Fig 1). *S*. *aureus* carriage increased with age: 11.3% (n = 38) in nurseries, 30.1% (n = 56) in DCCs and 50.0% (n = 29) in primary school (with a peak at 9–13 years). The other three bacteria showed an opposite tendency. In the three age categories, the carriage rates were 48.8% (n = 164), 21.5% (n = 40) and 6.9% (n = 4) for *S*. *pneumoniae*; 60.1% (n = 202), 37.6% (n = 70) and 15.5% (n = 9) for *M*. *catarrhalis*; 34.2% (n = 115), 19.9% (n = 37) and 0.0% (n = 0) for *H*. *influenzae*.

**Fig 2:**
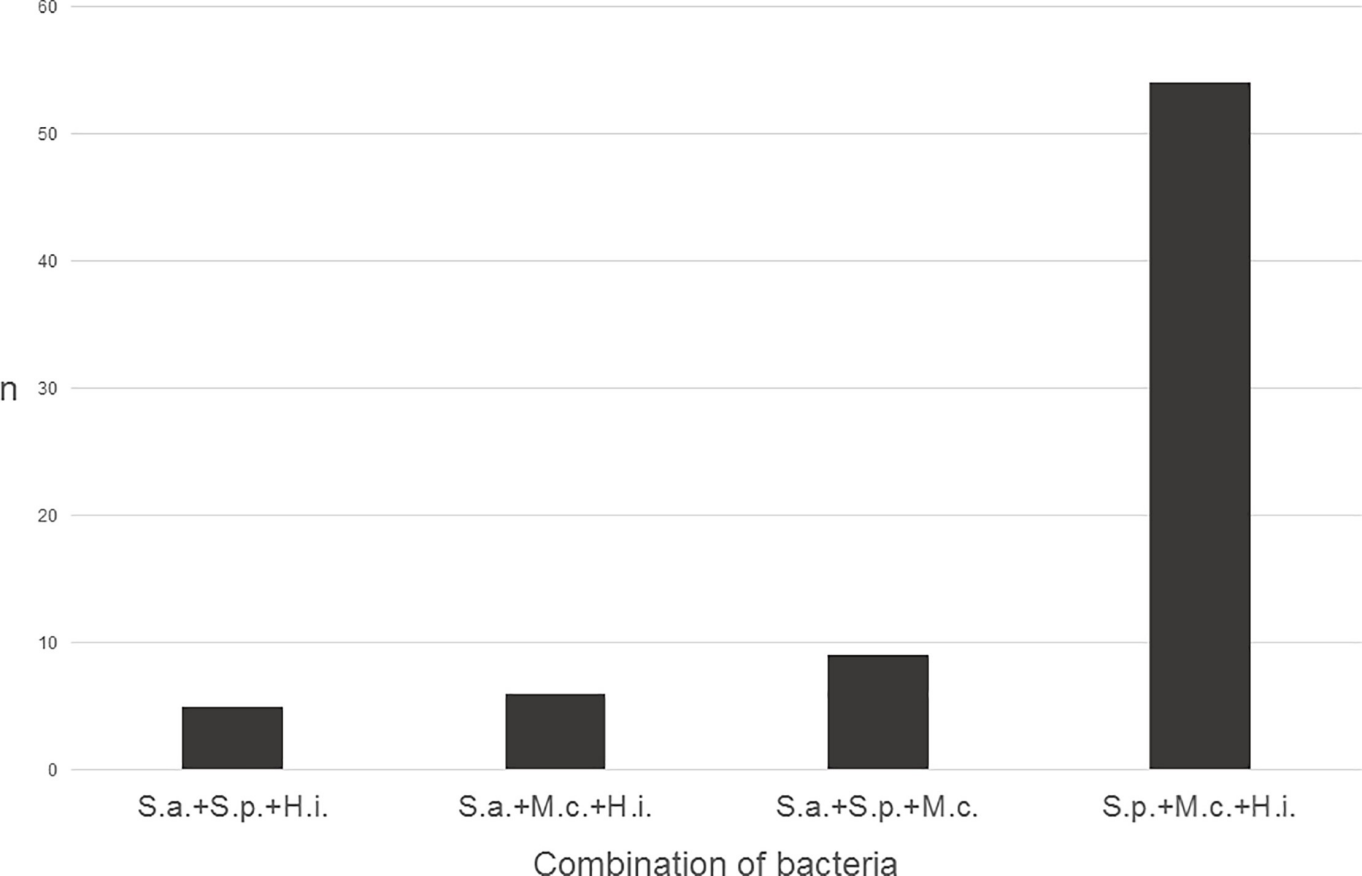
Carriage rates of three bacterial species together.

Table 1 shows the carriage rates of single or multiple pathogens related to age. It is clear from the data that multiple carriage occurs more frequently without *S*. *aureus*. This becomes most obvious if we look at triple carriage (Fig 2). The negative association was statistically significant between *S*. *aureus* and *S*. *pneumoniae* or *M*. *catarrhalis*, and it was nearly significant with *H*. *influenzae* (Table 2). On the other hand, the co-carriage of *S*. *pneumoniae*-*H*. *influenzae*, *S*. *pneumoniae*-*M*. *catarrhalis* and *H*. *influenzae*-*M*. *catarrhalis* was positively associated. No correlation was found, however, between VT pneumococci and the other three species.

**Table 1:** Colonisation rates of the four respiratory pathogens alone and in combination in the three different age groups.

	Nurseries (1-3y) n = 336		DCCs (3-6y) n = 186		Primary school (6-13y) n = 58	
Non-carriers	54	(16.1%)	60	(32.3%)	24	(41.4%)
Carriers	282	(83.9%)	126	(67.7%)	34	(58.6%)
Carriers of one pathogen	109	(32.4%)	71	(38.2%)	28	(48.3%)
*S*. *aureus*	10	(3.0%)	30	(16.1%)	24	(41.4%)
*S*. *pneumoniae*	26	(7.7%)	9	(4.8%)	1	(1.7%)
*M*. *catarrhalis*	54	(16.1%)	28	(15.1%)	3	(5.2%)
*H*. *influenzae*	19	(5.7%)	4	(2.2%)	0	(0.0%)
Carriers of two pathogens	111	(33.0%)	38	(20.4%)	4	(6.9%)
*S*. *pneumoniae* + *S*. *aureus*	2	(0.6%)	3	(1.6%)	0	(0.0%)
*S*. *pneumoniae + M*. *catarrhalis*	61	(18.2%)	8	(4.3%)	1	(1.7%)
*S*. *pneumoniae + H*. *influenzae*	17	(5.1%)	5	(2.7%)	0	(0.0%)
*M*. *catarrhalis + H*. *influenzae*	20	(6.0%)	9	(4.8%)	0	(0.0%)
*S*. *aureus + M*. *catarrhalis*	8	(2.4%)	10	(5.4%)	3	(5.2%)
*S*. *aureus + H*. *influenzae*	3	(0.9%)	3	(1.6%)	0	(0.0%)
Carriers of three pathogens	60	(17.9%)	12	(6.5%)	2	(3.4%)
*M*. *catarrhalis + H*. *influenzae + S*. *pneumoniae*	47	(14.0%)	7	(3.8%)	0	(0.0%)
*S*. *pneumoniae* + *S*. *aureus + M*. *catarrhalis*	6	(1.8%)	1	(0.5%)	2	(3.4%)
*S*. *pneumoniae* + *S*. *aureus + H*. *influenzae*	3	(0.9%)	2	(1.1%)	0	(0.0%)
*S*. *aureus + M*. *catarrhalis + H*. *influenzae*	4	(1.2%)	2	(1.1%)	0	(0.0%)
Carriers of four pathogens	2	(0.6)	5	(2.7%)	0	(0.0%)

**Table 2:** Statistical associations of co-carriage between the four species.

	*S*. *aureus*		p-value	*H*. *influenzae*		p-value	*M*. *catarrhalis*		p-value
	Carriers n (%)	Non-carriers n (%)		Carriers n (%)	Non-carriers n (%)		Carriers n (%)	Non-carriers n (%)	
*S*. *pneumoniae* carriers n = 208	26 (12.5)	182 (87.5)	**<0.001 (S)**	88 (42.3)	120 (57.7)	**<0.001 (S)**	140 (67.3)	68 (32.7)	**<0.001** **(S)**
*S*. *pneumoniae* non-carriers n = 372	97 (26.1)	275 (73.9)		64 (17.2)	308 (82.8)		141 (37.9)	231 (62.1)	
VT* *S*. *pneumoniae* carriers n = 13	2 (15.4)	11 (84.6)	0.669 (NS)	6 (46.2)	7 (53.8)	0.780 (NS)	9 (69.2)	4 (30.8)	1.000 (NS)
NVT *S*. *pneumoniae* carriers n = 195	24 (12.3)	171 (87.7)		82 (42.1)	113 (57.9)		131 (67.2)	64 (32.8)	
*S*. *aureus* carriers n = 123	-	-	-	24 (19.5)	99 (80.5)	0.065 (NS)	43 (35.0)	80 (65.0)	**<0.001 (S)**
*S*. *aureus* non-carriers n = 457	-	-		128 (28.0)	329 (72.0)		238 (52.1)	219 (47.9)	
*H*. *influenzae* carriers n = 152	-	-	-	-	-	-	96 (63.2)	56 (36.8)	**<0.001 (S)**
*H*. *influenzae* non-carriers n = 428	-	-		-	-		185 (43.2)	243 (56.8)	

*VT = PCV13 type

### Risk factors

The correlation between various risk factors and carriage rates were analysed individually in each age group (S1 Table), and also combined data were investigated (Table 3). Data were based on 576 questionnaires, as no answers were obtained from four children’s parents in the nursery group. To demonstrate relationship between risk factors and carriage, a p-value was calculated by the Fisher’s exact test with a threshold value of 0.05.

**Table 3:** Cumulative table of correlation between carriage and possible risk factors.

Risk factor	Pathogen	Screened children (1-13y) n = 576*		p-value
		Carriers (%)	NCs (%)	
Gender male	*S*. *p*. *S*. *a*. *M*. *c*. *H*. *i*.	101 (35.1) 68 (23.6) 130 (45.1) 80 (27.8)	187 (64.9) 220 (76.4) 158 (54.9) 208 (72.2)	0.794 (NS) 0.222 (NS) 0.156 (NS) 0.393 (NS)
Gender female	*S*. *p*. *S*. *a*. *M*. *c*. *H*. *i*.	105 (36.5) 55 (19.1) 148 (51.4) 70 (24.3)	183 (63.5) 233 (80.9) 140 (48.6) 218 (75.7)	
Having siblings	*S*. *p*. *S*. *a*. *M*. *c*. *H*. *i*.	129 (34.2) 90 (23.9) 173 (45.9) 91 (24.1)	248 (65.8) 287 (76.1) 204 (54.1) 286 (75.9)	0.315 (NS) **0.043 (S)** 0.136 (NS) 0.163 (NS)
Not having siblings	*S*. *p*. *S*. *a*. *M*. *c*. *H*. *i*.	77 (38.7) 33 (16.6) 105 (52.8) 59 (29.6)	122 (61.3) 166 (83.4) 94 (47.2) 140 (70.4)	
Antibiotic exposure in the past two weeks	*S*. *p*. *S*. *a*. *M*. *c*. *H*. *i*.	49 (34.5) 16 (11.3) 77 (54.2) 42 (29.6)	93 (65.5) 126 (88.7) 65 (45.8) 100 (70.4)	0.763 (NS) **0.001 (S)** 0.122 (NS) 0.272 (NS)
No antibiotics	*S*. *p*. *S*. *a*. *M*. *c*. *H*. *i*.	157 (36.2) 107 (24.7) 201 (46.3) 108 (24.9)	277 (63.8) 327 (75.3) 233 (53.7) 326 (75.1)	
Passive exposure to smoking	*S*. *p*. *S*. *a*. *M*. *c*. *H*. *i*.	51 (29.0) 43 (24.4) 85 (48.3) 42 (23.9)	125 (71.0) 133 (75.6) 91 (51.7) 134 (76.1)	**0.030 (S)** 0.270 (NS) 1.000 (NS) 0.471 (NS)
No passive exposure to smoking	*S*. *p*. *S*. *a*. *M*. *c*. *H*. *i*.	155 (38.8) 80 (20.0) 193 (48.3) 108 (27.0)	245 (61.3) 320 (80.0) 207 (51.8) 292 (73.0)	
Total number of carriers and NCs	*S*. *p*. *S*. *a*. *M*. *c*. *H*. *i*.	206 (35.8) 123 (21.4) 278 (48.3) 150 (26.0)	370 (64.2) 453 (78.6) 298 (51.7) 426 (74.0)	

*: Data of four children attending nursery were missing

NC: non-carrier

*M*. *catarrhalis* was the only bacterium, for which carriage was clearly not influenced by any of the risk factors. Regarding *S*. *aureus* carriage, statistically significant positive associations were found with male gender (only in primary school group) and having siblings (combined data), and a negative association with previous antibiotic exposure. The latter was also found for *H*. *influenzae* carriage, but only in the DCC group. Finally, passive smoking was negatively associated with *S*. *pneumoniae* carriage.

### Serotyping results

#### S. pneumoniae

In this survey 208 carriers were detected, however, one child each in the nursery and DCC group carried two different serotypes, resulting 210 *S*. *pneumoniae* isolates. The PCV13 vaccination rate was high, based on the questionnaires: 83.4% in nurseries, 81.7% in DCCs, 44.8% in primary school. As expected, PPV23 vaccination rates were lower (as it is an optional vaccination, and only suitable for older children): 6.9%, 8.6% and 5.2%, respectively. PCV13 serotypes were hardly present in the three groups: 4.8% (19F, 19A), 9.8% (19F, 9V) and 25.0% (7F), while PPV23 serotypes were more prevalent: 34.5% (15B, 11A, 10A, 9N, 33F, 22F), 39.0% (11A, 15B, 10A, 17F) and 0.0% (Table 4).

**Table 4:** PCV13, PPV23 and NVT serotype distribution among the three groups.

Serotype	Nurseries (1-3y) n = 165 (%)	DCCs (3-6y) n = 41 (%)	Primary school (6-13y) n = 4 (%)
PCV13 serotypes			
7F	0 (0.0)	0 (0.0)	1 (25.0)
9V	0 (0.0)	1 (2.4)	0 (0.0)
19A	1 (0.6)	0 (0.0)	0 (0.0)
19F	7 (4.2)	3 (7.3)	0 (0.0)
PPV23 serotypes			
9N	5 (3.0)	0 (0.0)	0 (0.0)
10A	9 (5.5)	2 (4.9)	0 (0.0)
11A	11 (6.7)	11 (26.8)	0 (0.0)
15B	28 (17.0)	2 (4.9)	0 (0.0)
17F	0 (0.0)	1 (2.4)	0 (0.0)
22F	1 (0.6)	0 (0.0)	0 (0.0)
33F	3 (1.8)	0 (0.0)	0 (0.0)
NVT serotypes			
6C	3 (1.8)	4 (9.8)	0 (0.0)
15A	10 (6.1)	0 (0.0)	0 (0.0)
15C	12 (7.3)	3 (7.3)	0 (0.0)
21	3 (1.8)	0 (0.0)	0 (0.0)
23A	14 (8.5)	0 (0.0)	0 (0.0)
23B	4 (2.4)	4 (9.8)	1 (25.0)
24F	19 (11.5)	0 (0.0)	0 (0.0)
31	0 (0.0)	3 (7.3)	0 (0.0)
34	5 (3.0)	1 (2.4)	0 (0.0)
35B	7 (4.2)	3 (7.3)	0 (0.0)
35F	16 (9.7)	1 (2.4)	0 (0.0)
37	0 (0.0)	0 (0.0)	2 (50.0)
38	4 (2.4)	0 (0.0)	0 (0.0)
NT	3 (1.8)	2 (4.9)	0 (0.0)
Total PCV13	8 (4.8)	4 (9.8)	1 (25.0)
Total PPV23	57 (34.5)	16 (39.0)	0 (0.0)
Total NVT	100 (60.6)	21 (51.2)	3 (75.0)

n: number of isolates

**Fig 3:**
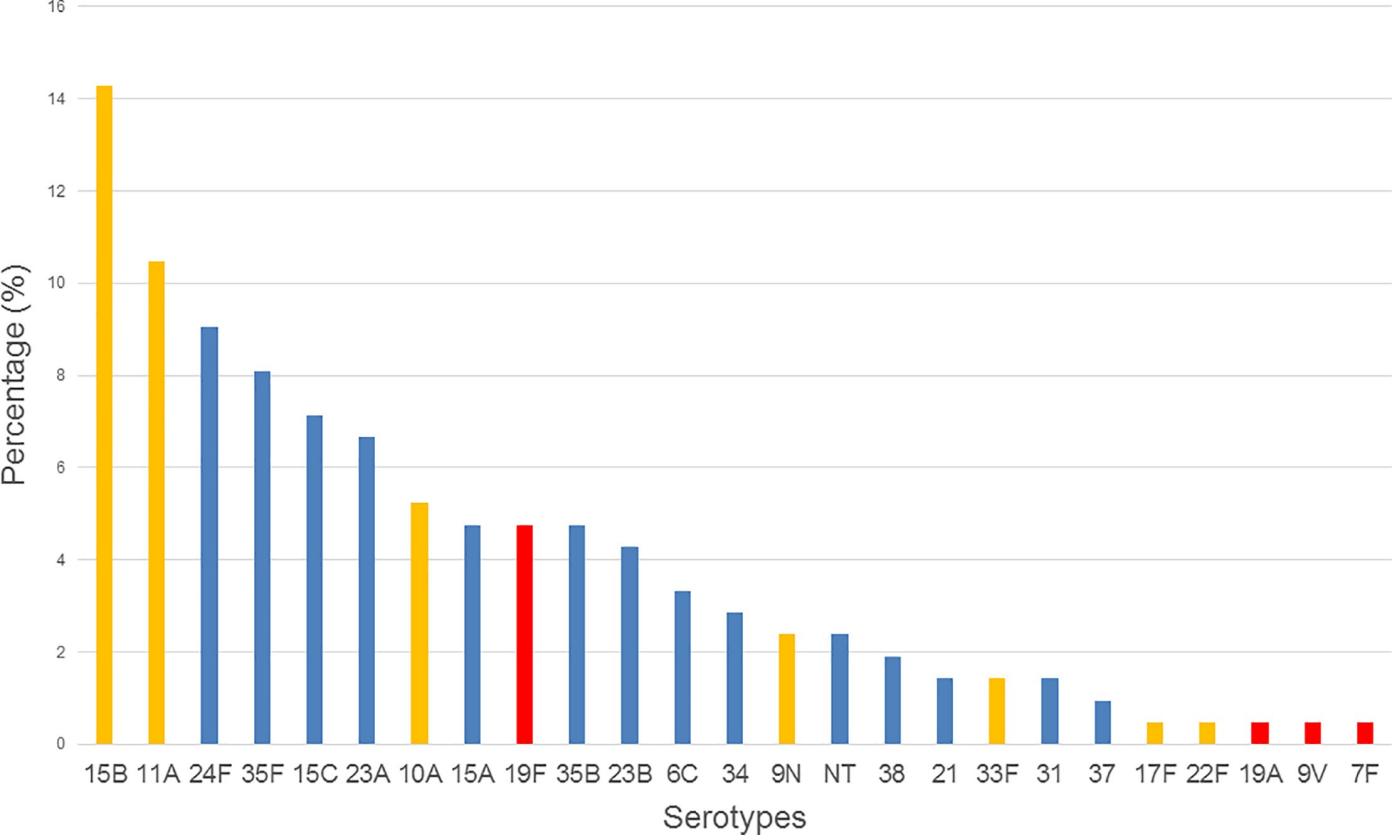
Summarized serotype distribution of the pneumococci from this study (n = 210). Red bars, PCV13 serotypes; Yellow bars, PPV23 serotypes; blue bars, NVT serotypes.

Due to the largest number of isolates (n = 165), the group of nurseries had the highest diversity with 20 different serotypes. In DCCs (n = 41) and in primary schools (n = 4), 14 and 3 serotypes were identified, respectively. The summarized serotype distribution can be seen in Fig 3. Serotype 15B was the leading type in the nurseries, while 11A was most prevalent in the DCCs. Serotypes 37 and 7F were only found in primary school. An increasing prevalence with age was observed in case of serotype 23B (2.4% in nurseries, 9.8% in DCCs and 25.0% among school children), serotype 11A (6.7% in nurseries vs. 26.8% in DCCs) and 6C (1.8% vs. 9.8%), while 15B and 24F showed decreasing tendencies (17.0% vs. 4.9% and 11.5% vs. 0.0%, respectively).

#### H. influenzae

We did not isolate any *H*. *influenzae* from primary school children. The serotype distribution among children attending DCCs (n = 37) was the following: 89.2% (n = 33) NTHi and 10.8% (n = 4) f; among children attending nurseries (n = 115): 95.7% (n = 110) NTHi, 2.6% (n = 3) f and 1.7% e (n = 2). Type b was not found throughout the study.

#### M. catarrhalis

Among primary school children, we obtained only 9 isolates and all of them belonged to serotype A. Serotype A was also most prevalent in DCCs (48/70, 68.6%) and nurseries (180/202, 89.1%). In the latter two age groups, serotype B was represented in 18.6% and 9.4%, and serotype C in 2.9% and 1.0%, respectively. The rest (10.0% versus 0.5%) was non-typeable.

### Antibiotic susceptibility

#### S. aureus

High penicillin resistance (73.7%-82.8%) was common in the three groups, but none of the isolates were resistant to oxacillin (Table 5). The absence of MRSA was confirmed by *mecA* negativity and cefoxitin screening. After penicillin, the highest resistance rate was measured for erythromycin, but it was only 12.2%. Vancomycin sensitivity was 100.0%. We found only one ciprofloxacin resistant isolate (MIC = 2 mg/L), which was obtained from a child in the nursery group. There were three mupirocin resistant isolates, all of them from children attending DCCs. This corresponds to a 2.4% prevalence in total, and 5.4% among the DCCs. For all three resistant isolates the mupirocin MIC value was >1024 mg/L. Concordantly, all of them possessed the *mupA* gene and none of them had point mutations in the *ileS* gene.

**Table 5:** Summarized antibiotic susceptibility data of the bacterial isolates from this study.

Bacteria (n) and antibiotics	MIC range (mg/L)	MIC50 (mg/L)	MIC90 (mg/L)	S (%)	I (%)	R (%)
*S*. *pneumoniae* (n = 210)						
Penicillin	<0.015–2	0.03	0.25	86.7	13.3	0.0
Cefotaxime	<0.004–1	0.03	0.125	96.2	3.8	0.0
Imipenem	<0.004–0.5	0.015	0.06	100.0	0.0	0.0
Erythromycin	<0.06->256	0.125	32	82.9	0.0	17.1
Clindamycin	<0.5->128	<0.5	<0.5	91.9	0.0	8.1
Levofloxacin	<0.5–2	1	2	100.0	0.0	0.0
Moxifloxacin	<0.03–0.5	0.25	0.25	100.0	0.0	0.0
Vancomycin	<0.125–1	0.5	1	100.0	0.0	0.0
*S*. *aureus* (n = 123)						
Penicillin	<0.03–256	8	64	22.8	0.0	77.2
Oxacillin	0.06–0.5	0.25	0.5	100.0	0.0	0.0
Tetracycline	0.25->4	0.5	1	98.4	0.0	1.6
Erythromycin	0.19->256	0.19	>256	87.8	0.0	12.2
Clindamycin	0.094–1	0.094	0.25	99.2	0.0	0.8
Gentamicin	0.5–4	1	1	95.1	0.0	4.9
Ciprofloxacin	0.25–2	0.5	1	99.2	0.0	0.8
Mupirocin	<0.03->1024	0.06	0.06	97.6	0.0	2.4
Vancomycin	1–2	1	2	100.0	0.0	0.0
*H*. *influenzae* (n = 152)						
Ampicillin	0.25->8	1	1	93.4	0.0	6.6
Amoxicillin-clavulanic acid	0.125–0.75	0.5	0.5	100.0	0.0	0.0
Cefotaxime	0.008–0.06	0.015	0.06	100.0	0.0	0.0
Levofloxacin	<0.004–0.125	0.008	0.015	100.0	0.0	0.0
Moxifloxacin	<0.004–0.5	0.03	0.06	100.0	0.0	0.0
Erythromycin	<0.25–32	8	16	96.7	0.0	3.3
Azithromycin	0.06–8	2	4	90.8	0.0	9.2
Trimethoprim-sulfamethoxazole	0.008->32	0.03	>32	85.5	0.0	14.5
*M*. *catarrhalis* (n = 281)						
Amoxicillin-clavulanic acid	<0.06–0.38	0.094	0.25	100.0	0.0	0.0
Cefotaxime	<0.004–1	0.25	0.5	100.0	0.0	0.0
Levofloxacin	0.015–0.5	0.03	0.06	98.2	0.0	1.8
Moxifloxacin	0.06–0.25	0.06	0.125	100.0	0.0	0.0
Erythromycin	<0.125–16	0.25	0.25	96.1	2.5	1.4
Azithromycin	<0.06–8	0.06	0.06	97.9	0.4	1.8
Trimethoprim-sulfamethoxazole	0.015–2	0.125	0.25	98.9	0.4	0.7

#### S. pneumoniae

No penicillin resistant isolates were found in this study, but an overall 13.3% of the strains belonged to the intermediate category (0.12–2 mg/L; Table 5). Intermediate resistance was much higher among the DCC isolates (26.8%) than among the nursery isolates (9.7%), and also one out of the four school isolates (25.0%) showed intermediate resistance. Erythromycin resistance was also higher in the DCC group (29.3%) than in the nursery group (14.5%). Pneumococci were 100.0% susceptible to fluoroquinolones and vancomycin. More details about the 206 isolates from nurseries and DCCs are available from our previous publication [31].

The different resistance levels in the three age groups could be explained by the different serotype prevalence. For example, serotype 11A, which was more prevalent in the DCC group (Table 4), contributed significantly to erythromycin resistance, 13/22 (59.1%) of 11A isolates being resistant (mostly M phenotype).

#### H. influenzae

Ampicillin resistance was 7.8% among the nursery isolates and 2.7% in the DCC group. All 10 amp^R^ strains had an MIC value >8mg/L, but all were susceptible to amoxicillin/clavulanic acid (Table 5). Beta-lactamase production was confirmed by a positive nitrocefin disc test in every case. Only a small proportion of the isolates showed macrolide resistance (3.3% to erythromycin). The highest resistance level was measured for trimethoprim-sulfamethoxazole (14.5% in average; 11.3% in the nurseries and 24.3% in the DCCs). Of importance, all nine serotypeable isolates were fully susceptible to all the tested antibiotics.

#### M. catarrhalis

All *M*. *catarrhalis* isolates were remarkably susceptible to antibiotics. Full susceptibility was measured to amoxicillin/clavulanic acid, cefotaxime and moxifloxacin (Table 5). Among the total of 281 strains, only five were levofloxacin resistant, deriving from nurseries (however, all of them had an MIC of only 0.25 or 0.5 mg/L). Non-susceptibility to macrolides and trimethoprim-sulfamethoxazole was also below 5% and always with low MIC values.

### Clonality

The complete PFGE dendrograms of all NTHi *H*. *influenzae* isolates are shown in S1 Fig. Here, we describe only the clonality of the typeable strains.

**Fig 4:**
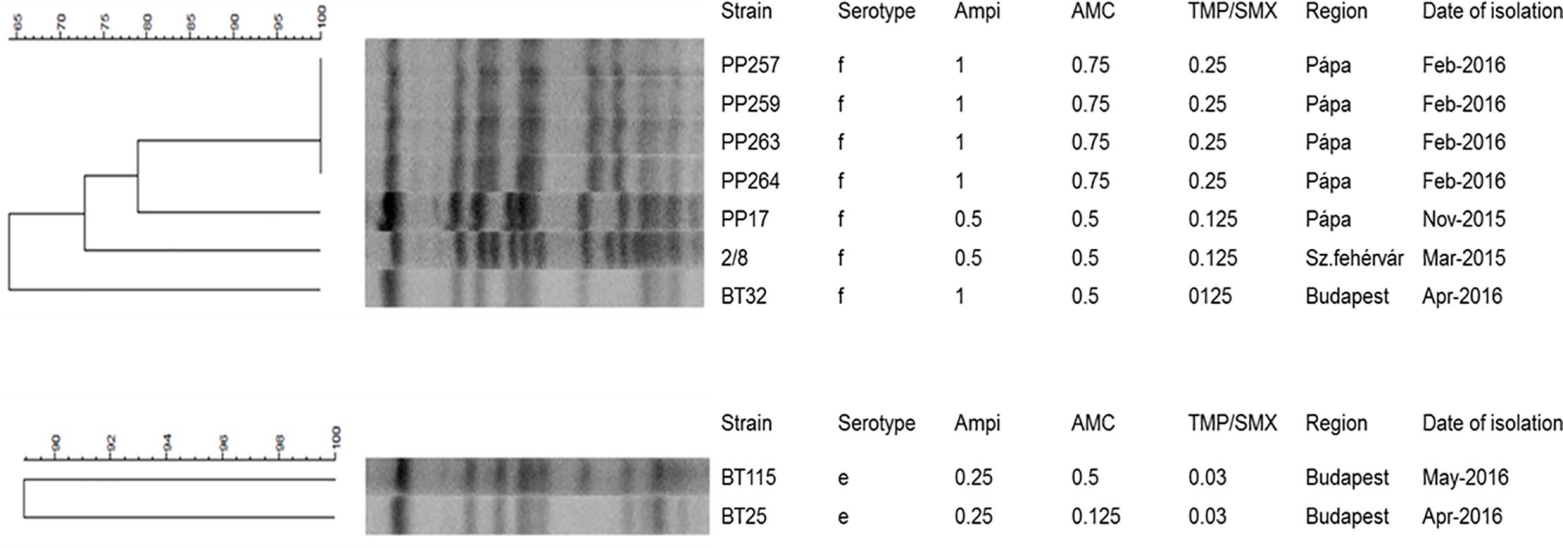
PFGE pattern of the serotype f and e *H*. *influenzae* isolates.

Based on their restriction patterns, four of the seven *H*. *influenzae* serotype f isolates—originating from the same DCC—were indistinguishable (Fig 4). The other three, collected at three different institutions in three different cities, showed 4–6 fragment differences (i.e., “possibly related”, according to Tenover’s criteria [32]). The two serotype e isolates also shared a high level of similarity (one band difference, Fig 4). On the other hand, NTHi isolates showed much higher variability, forming several epidemiologically unrelated smaller clusters, typically consisting of 2–8 isolates (S1 Fig).

The *S*. *aureus* samples generally showed high genetic diversity, comprising of several distinct types (S2 Fig). However, there were some bigger clusters that contained closely related isolates (≤ 3 bands difference), often originating from different cities and different institutes.

**Fig 5:**
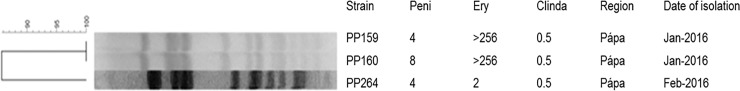
PFGE pattern of the three mupirocin resistant *S*. *aureus* isolates.

Regarding the three mupirocin resistant strains, two–deriving from the same DCC–showed 100.0% identity based on the PFGE picture (Fig 5). They also shared very similar antibiotic resistance patterns. The third isolate (PP264) had a slightly diverging banding pattern compared to the previous two and it was only intermediate resistant to erythromycin and originated from a different DCC.

**Fig 6:**
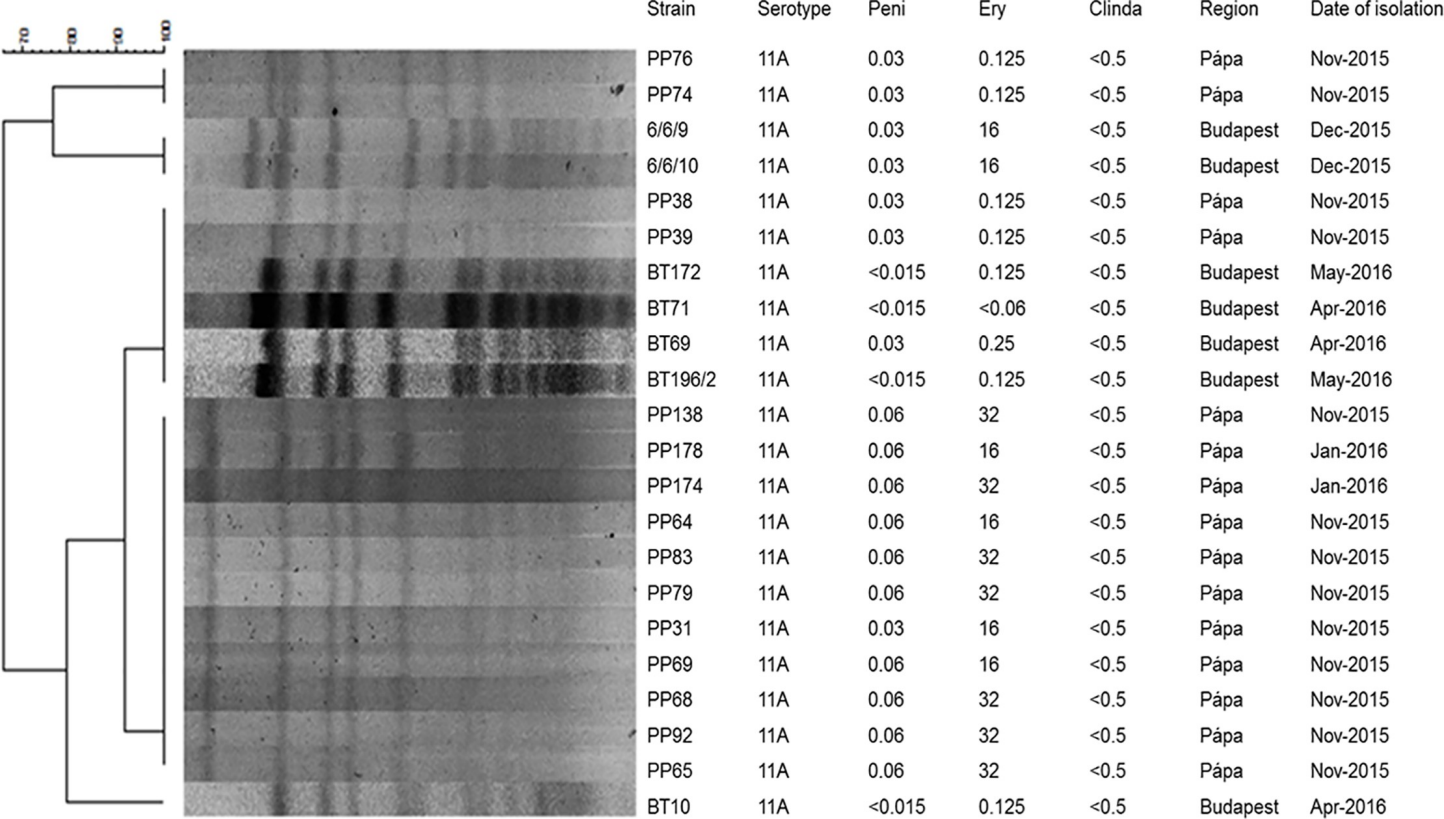
PFGE dendrogram of the serotype 11A pneumococcal isolates from this study.

For pneumococci, the genetic relatedness of serotypes 3 and 19F was investigated in our previous study [31]. Here we show the PFGE picture of serotype 11A, which was most prevalent in the DCCs (Fig 6). As it can be seen, most of them are closely related (≤ 3 bands difference), but especially the clonality of the macrolide resistant isolates (M type) can be emphasized.

## Discussion

In this study, we surveyed the nasal colonization of four potentially pathogenic bacteria in three different age groups over a 15-month time period. To our best knowledge, this is the first study from Hungary about the asymptomatic carriage of *H*. *influenzae* and *M*. *catarrhalis*, but our workgroup has published data on pneumococcal and *S*. *aureus* carriage in the last ten years.

The observed pneumococcal carriage rates correspond well with international data. We measured the highest rate (48.8%) of pneumococcal carriage in nurseries, 21.5% in DCCs and only 6.9% among primary school children. In contrast, the *S*. *aureus* carriage rate was 11.3% in the first age group, 30.1% in the DCCs and the highest among primary school children (50.0%). These figures almost exactly coincide with the results of Bogaert *et al*. and Sollid *et al*. [34–36]. They have shown that following the rapid and intensive colonisation with *S*. *aureus* right after birth, carriage drops to around 12% by 14 months of age. Later it starts increasing again to about 20–30% for DCC children and peaks at around 40–50% in school-age children. Regarding pneumococcal carriage, according to previous publications, it peaks at 2–3 years of age at around 50–60% and from that point it linearly decreases; in DCC children it is typically around 20–30%, while in school children it falls below 10% [36, 37]. In the study of Žemličková et al, when conducting a similar carriage survey for the same four pathogens in the Czech Republic in 2004–2005, the same age-related tendency was registered for all four bacteria [38].

We can also compare the current results with our previous findings. Between 2009 and 2011, we had screened 878 healthy children attending DCCs in 16 different cities of Hungary and we obtained an *S*. *aureus* carriage rate of 21.3% [18]. In 2012 we have conducted a survey, screening all children at the DCCs of one town (n = 1390) and here we found a higher carriage rate (34.1%) [39]. In the current study, the carriage of DCC children was 30.1%.

We could follow pneumococcal carriage along with the changing vaccination situation over the last few years [40]. Although there has been a huge serotype rearrangement in these years, the carriage rate of children from DCCs remained nearly the same: 34.1% in the pre-PCV7 era and 32.5% in the post-PCV7-pre-PCV13 period. Remarkably the carriage rate has dropped by now (i.e. the post-PCV13 era) to 21.5% in the same age group.

All children in this study were vaccinated with Hib, as the oldest children were born in 2003 and the vaccination rate in that year was 99.8–100.0% [41]. The difference between the three groups was their PCV13 vaccination status. The PCV13 vaccination rates based on the parents’ answers were 83.4% in the nurseries, 81.7% in the DCCs and 44.8% among school children. It is known, however, from the national surveillance reports, that these figures must have been even higher (see Introduction). The children of this study were born between 2003 and 2015. A certain cohort in the primary school group was too old to be vaccinated, but the oldest children in the DCCs were born in 2009, and PCV vaccination coverage was already 89.1% for them. For children born in 2014 and 2015, the rates were 98.5% and 99.9%, respectively [42, 43].

The carriage rates of *H*. *influenzae* and *M*. *catarrhalis* observed in this study are also consistent with data from the literature. For instance, 35.5% *H*. *influenzae* carriage was reported among 0–6 years old children in Japan [44] and 15.6% among preschool children (aged 5–6 years) in Turkey [45] and 24.9% in DCC children in the Czech Republic [38]. Our corresponding figures are 34.2% (nurseries) and 19.9% (DCCs), respectively. Similar to other countries where Hib had been implemented in the NIP, serotype b was not found among our isolates [44, 46]. For example, only NTHi strains were detected in Italy among children <6 years old [46]. In addition, *H*. *influenzae* infections shifted to older age groups and towards NTHi, due to worldwide vaccination [10, 47]. Besides NTHi, only 3 isolates each of serotype e and f were found in the Czech study [38]. The overwhelming majority of our strains were also NTHi, and only a few serotype f and e isolates were identified.

*M*. *catarrhalis* carriage also showed a clear decreasing tendency in our study, from 60.1% in nurseries to 37.6% in DCCs and further to 15.5% among school children. Very similar rates were reported from Japan, where 58.1% was observed in children <6 years old [44] and Turkey, where the carriage rate was 23.9% in 6–10 years old children [48]. We observed a dominance of serotype A (overall 84.3%), followed by type B (11.4%), type C (1.4%), and finally 2.8% of the strains were NT. This is also in good correlation with previously published data [25, 49]. In a recent review by Blakeway *et al*., the following serotype prevalence was summarised, based on several other studies: A (60–75%) > B (20–30%) > C (2–6%) > non-typeable (ca. 5%) [49].

Several European publications have reported carriage data from adults as well. *S*. *aureus* carriage was 27.3% in Norwegian adults [50] and 37.4% in Switzerland [51]. According to a recent German study, it still remained 28.5% in the elderly (≥65 years) [52]. In this latter study, the carriage rate was 1.9% for *H*. *influenzae* and no *S*. *pneumoniae* were found. A very low prevalence of *H*. *influenzae*, *S*. *pneumoniae* and *M*. *catarrhalis* carriage in adults was also described by Swedish authors [53].

Besides eliminating vaccine-type (VT) pneumococci, PCVs influence the distribution of other bacteria as well. These changes include elevated *S*. *aureus*, *H*. *influenzae* and *M*. *catarrhalis* carriage rates in children, according to other studies. A rapid rise in *H*. *influenzae* and Moraxella carriage was observed in Japan among DCC children already one year post-PCV, despite the fact that vaccination was not fully implemented in the country [44]. A group in the Netherlands has conducted a long-term follow-up to check the effect of PCVs on other bacteria. They found that–both in 11 months old, 24 months old children and their parents–the carriage of the other three species had significantly increased when testing 4.5 years post-PCV, but their ratio started declining again 7 years post-PCV [54, 55]. Moreover, *H*. *influenzae* and *M*. *catarrhalis* were more frequently isolated from the nasopharynx of children diagnosed with acute otitis media in the USA, a few years after PCV7 was introduced [56].

We cannot draw conclusions concerning the indirect effects of PCV13 based on this study as we do not have base-line data on *H*. *influenzae* and *M*. *catarrhalis* carriage among children from the pre-PCV13 era in Hungary. There was no correlation in this study between PCV13 vaccination status and *H*. *influenzae* or *M*. *catarrhalis* carriage in any of the age groups (S1 Table). Furthermore, although there was a positive association between the carriage of S. *pneumoniae* and either of the other two species, it was independent of the fact, whether it was a VT or NVT pneumococcus (Table 2). The *S*. *aureus* rate has increased from 21.3% to 30.1% among DCC children, compared to our study from 2009–2011 (18). The direct effect is indisputable, mirrored by the low ratio of PCV13 serotypes (6.2% in average, Table 4).

Various studies have shown that *S*. *pneumoniae* and *S*. *aureus* interfere with one another in nasopharyngeal colonization [57–59]. There are several possible explanations for this: beside natural competition for the niche, the production of inhibitory H_2_O_2_ and other bacteriocins by the pneumococcus may play a role [58]. Reddinger *et al*. have performed an interesting experiment in mice: when artificially colonizing the airways of mice with the two bacteria, and then infecting them with influenza virus, only the pneumococcus was able to disperse from the biofilm state and cause pneumonia. This indicated that the pathogenic potential of *S*. *aureus* was reduced in the presence of pneumococcus [60]. In the current study, the negative association was obvious. When summarising the results of all our previous carriage studies, examining altogether 2268 children attending DCCs, only 7.1% of the children proved to be double carriers of these two species [61]. Now, *S*. *aureus* was shown to have an interference not only with pneumococcus, but with *H*. *influenzae* and *M*. *catarrhalis* as well (Table 2 and Fig 2). This raises the question which factors are responsible for this repulsion from the side of *S*. *aureus*. On the other hand, we can conclude that carrying PCV13 pneumococci did not influence the *S*. *aureus* carriage rate: it was 12.3% among VT-carriers and 15.4% among NVT-carriers (Table 2).

As for the other risk factors, male gender has often been associated with *S*. *aureus* carriage [62, 63]. This was also found in one of our previous projects [39], but not in another [18]. In the current study, the association was only statistically significant among school-age children, but not for the combined data (S1 Table and Table 3). Carrying *S*. *aureus* has a higher likelihood in an environment where other *S*. *aureus* carriers are present (e.g. having siblings attending another community) [62, 63] as was documented also by us before [18] and now confirmed again. Recent antibiotic exposure can have a two-sided effect: it can facilitate the selection and spread of antibiotic resistant bacteria but can lead to the eradication of susceptible isolates [64–67]. In our survey, a negative correlation was found not just with *S*. *aureus*, but also with *H*. *influenzae* carriage (S1 Table and Table 3).

Both active and passive smoking might represent a higher risk for bacterial colonization and disease development according to some researches [68, 69]. However, the opposite association has also been described before, for instance in a Belgian study, where parental smoking negatively influenced pneumococcal colonization in 6–30 months old infants [70]. Here, we also found a negative association between *S*. *pneumoniae* carriage and exposure to passive smoking (Table 3). Interestingly, we have found earlier that a much higher percentage of the serotype 19A carriers were exposed to passive smoking, although this was not statistically significant [40].

The dynamic changes in pneumococcal serotype distribution among DCC children over the last few years were discussed in our previous work [31]. Most importantly, a clear decrease in PCV13 serotypes could be observed in parallel to the increasing PCV vaccination rates. It fell from an initial 57.3% in the pre-PCV7 era to 32.5% in the post-PCV7-pre-PCV13 era (40), and further to 6.2% in the post-PCV13 era (current report).

Out of the 13 PCV13 type isolates in total, however, 10 were of serotype 19F, while 19A, 9V and 7F were detected in one case each (Table 4). Serotype 3, 6A and 6B or 23F have completely vanished by now. Among the non-PCV serotypes, 15B was dominant in the nurseries (17.0%) and 11A in the DCCs (26.8%). Both are part not only of the PPV23 vaccine, but also of the future 20-valent PCV as well, highlighting the importance of an extended serotype coverage in vaccination. Furthermore, serotype 11A ranks among the top ten IPD causing serotypes according to the latest ECDC report [71].

The carried isolates were more sensitive to antibiotics in case of all four bacterial species, compared to clinical isolates in Hungary, from the same time period. These latter data are available online, in Hungarian, reported annually by the National Bacteriological Surveillance Management Team of the National Public Health Center [72]. In case of *S*. *aureus* and *S*. *pneumoniae*, this difference has been observed also in our previous studies [18, 39, 40, 73].

For instance, the erythromycin resistance of clinical *S*. *aureus* isolates (from outpatients) in 2015 and 2016 –the time of our study–was 22–24%, while only 12.2% for our strains; or tetracycline resistance was 7–8% in outpatients, while only 1.6% in this study. Of note, penicillin resistance of the *S*. *aureus* isolates in this study was somewhat lower compared to our previous carriage projects [18, 39]. As we did not detect any MRSA isolates in this study, the ciprofloxacin resistance was accordingly low (0.8% only, compared to 8.2–8.5% among clinical isolates). According to our previous results, 0.8% of the carried isolates were methicillin-resistant [39]. The increasing pressure to prevent MRSA infections led to an increased use of mupirocin for nasal decolonization worldwide and as a consequence, mupirocin resistance emerged [74]. In this study we found three mupirocin resistant strains (2.4%). This is higher than among clinical isolates (1.1–1.6%) from the same time period in Hungary [72].

We did not observe any penicillin or levofloxacin resistant pneumococcal isolates (13.3% of the strains belonged to the penicillin intermediate category), whereas the resistance rates for these two drugs were 2.5–2.9% and 0.8–1.8% for the Hungarian clinical isolates, respectively [72]. The two Gram-negative species were basically more sensitive than the two Gram-positive ones. For *H*. *influenzae*, ampicillin resistance was 6.6% in this study, while it was twice as high for clinical isolates (13.7–13.8%). Similarly, whereas all our isolates were fully susceptible to amoxicillin-clavulanic acid, there was 5.3–6.3% resistance in case of the disease causing strains. In general, *β*-lactam resistance in this bacterium is mediated by the production of *β*-lactamases, but an altered penicillin-binding protein can also result in a lower affinity to *β*-lactams. These strains are called beta-lactamase negative ampicillin resistant (BLNAR) [75]. Only trimethoprim-sulfamethoxazole (TMP-SMX) resistance found in our study was comparable to the clinical situation: 14.5% in our study vs. 17–19% among clinical patients [72]. Regarding *M*. *catarrhalis*, we measured very low resistance percentages: 1.4% to erythromycin or 0.7% to TMP-SMX, versus ~11% and ~14% in clinical isolates [72].

In the above mentioned Turkish survey, antibiotic resistance of co-carried *S*. *pneumoniae*, *H*. *influenzae* and *M*. *catarrhalis* isolates was determined among 6–10 years old school children [48]. They measured higher rates of resistance compared to our study. In case of *S*. *pneumoniae*, 7% to penicillin, 42% to erythromycin and 34% to clindamycin, while our corresponding figures were 0%, 17.1% and 8.1%. Regarding TMP-SMX, higher rates were measured both for *H*. *influenzae* (28.6%) and *M*. *catarrhalis* (20.0%), compared to ours. All *M*. *catarrhalis* isolates were amoxicillin/clavulanic acid sensitive also in the Turkish study.

The existing Hib and PCV vaccines have the potential to partially overcome the antibiotic resistance problem, as resistance is serotype-linked. For instance, most invasive *H*. *influenzae* infections in India in the 1990s were caused by type b and in parallel increasing ampicillin and chloramphenicol resistance was observed [76]. PCVs were designed to include the most prevalent serotypes among invasive diseases, and some of these turned out to be associated with high resistance levels (such as 19A). The vaccine-driven serotype replacement in pneumococci has resulted the emergence of newer types, which, however, are typically more susceptible to antibiotics. For example, it was shown that the reduced incidence of invasive pneumococcal disease and otitis media (after the introduction of PCV7) was accompanied by a more moderate prescription of antibiotics in the USA between 2000 and 2010 [77]. Serotype replacement also appears in carriage, where antibiotic resistance is even lower. We suggest vaccination should be continued with the existing products, but we shall work on future vaccines (including those against *S*. *aureus* and *M*. *catarrhalis*) and keep on surveying isolates circulating in carriage.

The study has the following limitations: (i) the country was not fully covered geographically when collecting the specimens, (ii) we could not fully exclude that seasonality might have influenced colonisation, as different institutions were not visited evenly throughout the study period (and age seemed to be a stronger influencing factor) and (iii) virulence factors were not detected e.g. for *S*. *aureus*.

## Supporting information

S1 Table Correlation between carriage and possible risk factors in the three age groups. (DOCX)

S1 Fig PFGE dendrogram of the NTHi isolates (n = 141/143, two of them could not be digested by *SmaI*). (DOCX)

S2 Fig PFGE dendrogram of all *S*. *aureus* isolates from this study (n = 122/123, one of them could not be digested by *SmaI*). (DOCX)

S1 Raw Images The original *H*. *influenzae* PFGE gel pictures. (PDF)

S2 Raw Images The original *S*. *aureus* PFGE gel pictures. (PDF)

S3 Raw Images The original *S*. *pneumoniae* PFGE gel pictures. (PDF)

## Abbreviations

PCV: pneumococcal conjugated vaccine
Hib: *Haemophilus influenzae* type b
DCC: day-care centre
VT: vaccine type
MIC: minimum inhibitory concentration
BLAST: basic local alignment search tool
PFGE: pulsed-field gel electrophoresis
PPV: pneumococcal polysaccharide vaccine
NIP: national immunization programme
TMP-SMX: trimethoprim-sulfamethoxazole
