# Supplementary material for: Co-carriage of Staphylococcus aureus, Streptococcus pneumoniae, Haemophilus influenzae and Moraxella catarrhalis among three different age categories of children in Hungary
Source: PLoS One. 2020 Feb 7;15(2):e0229021. doi: 10.1371/journal.pone.0229021 (PMC7006921; doi:10.1371/journal.pone.0229021)
Supplement: S1 Table — (DOCX) [file pone.0229021.s001.docx]

| Risk factor | Patho-gen | Nurseries (1-3y) n=332* | | p-value | DCCs (3-6y)  n=186 | | p-value | Primary school (6-13y) n=58 | | p-value |
| --- | --- | --- | --- | --- | --- | --- | --- | --- | --- | --- |
|  |  | Carriers  (%) | NCs  (%) |  | Carriers  (%) | NCs  (%) |  | Carriers (%) | NCs  (%) |  |
| Gender male | *S. p.*  *S. a.*  *M. c.*  *H. i.* | 78 (49.1)  18 (11.3)  92 (57.9)  61 (38.4) | 81 (50.9)  141 (88.7)  67 (42.1)  98 (61.6) | 1.000 (NS)  1.000 (NS)  0.502 (NS)  0.132 (NS) | 20 (20.6)  29 (29.9)  34 (35.1)  19 (19.6) | 77 (79.4)  68 (70.1)  63 (64.9)  78 (80.4) | 0.859 (NS)  1.000 (NS)  0.454 (NS)  1.000 (NS) | 3 (9.4)  21 (65.6)  4 (12.5)  0 (0.0) | 29 (90.6)  11 (34.4)  28 (87.5)  32 (100.0) | 0.620 (NS)  **0.017 (S)**  0.717 (NS)  **-** |
| Gender female | *S. p.*  *S. a.*  *M. c.*  *H. i.* | 84 (48.6)  20 (11.6)  107 (61.8)  52 (30.1) | 89 (51.4)  153 (88.4)  66 (38.2)  121 (69.9) |  | 20 (22.5)  27 (30.3)  36 (40.4)  18 (20.2) | 69 (77.5)  62 (69.7)  53 (59.6)  71 (79.8) |  | 1 (3.8)  8 (30.8)  5 (19.2)  0 (0.0) | 25 (96.2)  18 (69.2)  21 (80.8)  26 (100.0) |  |
| Having siblings | *S. p.*  *S. a.*  *M. c.*  *H. i.* | 95 (49.0)  21 (10.8)  114 (58.8)  63 (32.5) | 99 (51.0)  173 (89.2)  80 (41.2)  131 (67.5) | 1.000 (NS)  0.728 (NS)  0.650 (NS)  0.483 (NS) | 31 (23.7)  43 (32.8)  51 (38.9)  28 (21.4) | 100 (76.3)  88 (67.2)  80 (61.1)  103 (78.6) | 0.330 (NS)  0.226 (NS)  0.621 (NS)  0.547 (NS) | 3 (5.8)  26 (50.0)  8 (15.4)  0 (0.0) | 49 (94.2)  26 (50.0)  44 (84.6)  52 (100.0) | 0.362 (NS)  1.000 (NS)  1.000 (NS)  - |
| Not having siblings | *S. p.*  *S. a.*  *M. c.*  *H. i.* | 67 (48.6)  17 (12.3)  85 (61.6)  50 (36.2) | 71 (51.4)  121 (87.7)  53 (38.4)  88 (63.8) |  | 9 (16.4)  13 (23.6)  19 (34.5)  9 (16.4) | 46 (83.6)  42 (76.4)  36 (65.5)  46 (83.6) |  | 1 (16.7)  3 (50.0)  1 (16.7)  0 (0.0) | 5 (83.3)  3 (50.0)  5 (83.3)  6 (100.0) |  |
| Vaccinated with Prevenar 13 | *S. p.*  *S. a.*  *M. c.*  *H. i.* | 139 (50.2)  33 (11.9)  163 (58.8)  96 (34.7) | 138 (49.8)  244 (88.1)  114 (41.2)  181 (65.3) | 0.302 (NS)  0.649 (NS)  0.452 (NS)  0.643 (NS) | 33 (21.7)  47 (30.9)  57 (37.5)  31 (20.4) | 119 (78.3)  105 (69.1)  95 (62.5)  121 (79.6) | 1.000 (NS)  0.683 (NS)  1.000 (NS)  0.816 (NS) | 1 (3.8)  13 (50.0)  3 (11.5)  0 (0.0) | 25 (96.2)  13 (50.0)  23 (88.5)  26 (100.0) | 0.620 (NS)  1.000 (NS) 0.495 (NS)  - |
| Not vaccinated with Prevenar 13 | *S. p.*  *S. a.*  *M. c.*  *H. i.* | 23 (41.8)  5 (9.1)  36 (65.5)  17 (30.9) | 32 (58.2)  50 (90.9)  19 (34.5)  38 (69.1) |  | 7 (20.6)  9 (26.5)  13 (38.2)  6 (17.6) | 27 (79.4)  25 (73.5)  21 (61.8)  28 (82.4) |  | 3 (9.4)  16 (50.0)  6 (18.8)  0 (0.0) | 29 (90.6)  16 (50.0)  26 (81.3)  32 (100.0) |  |
| Antibiotic exposure in the past two weeks | *S. p.*  *S. a.*  *M. c.*  *H. i.* | 46 (43.8)  7 (6.7)  65 (61.9)  41 (39.0) | 59 (56.2)  98 (93.3)  40 (38.1)  64 (61.0) | 0.239 (NS)  0.066 (NS)  0.632 (NS)  0.213 (NS) | 3 (9.7)  7 (22.6)  10 (32.3)  1 (3.2) | 28 (90.3)  24 (77.4)  21 (67.7)  30 (96.8) | 0.095 (NS)  0.394 (NS)  0.548 (NS)  **0.012 (S)** | 0 (0.0)  2 (33.3)  2 (33.3)  0 (0.0) | 6 (100.0)  4 (66.7)  4 (66.7)  6 (100.0) | 1.000 (NS)  0.670 (NS)  0.231 (NS)  - |
| No antibiotics | *S. p.*  *S. a.*  *M. c.*  *H. i.* | 116 (51.1)  31 (13.7)  134 (59.0)  72 (31.7) | 111 (48.9)  196 (86.3)  93 (41.0)  155 (68.3) |  | 37 (23.9)  49 (31.6)  60 (38.7)  36 (23.2) | 118 (76.1)  106 (68.4)  95 (61.3)  119 (76.8) |  | 4 (7.7)  27 (51.9)  7 (13.5)  0 (0.0) | 48 (92.3)  25 (48.1)  45 (86.5)  52 (100.0) |  |
| Passive exposure to smoking | *S. p.*  *S. a.*  *M. c.*  *H. i.* | 35 (44.9)  6 (7.7)  47 (50.3)  25 (32.1) | 43 (55.1)  72 (92.3)  31 (39.7)  53 (67.9) | 0.440 (NS)  0.310 (NS)  1.000 (NS)  0.785 (NS) | 14 (18.2)  26 (33.8)  34 (44.2)  17 (22.1) | 63 (81.8)  51 (66.2)  43 (55.8)  60 (77.9) | 0.372 (NS)  0.418 (NS)  0.128 (NS)  0.578 (NS) | 2 (9.5)  11 (52.4)  4 (19.0)  0 (0.0) | 19 (90.5)  10 (47.6)  17 (81.0)  21 (100.0) | 0.615 (NS)  1.000 (NS)  0.710 (NS)  - |
| No passive exposure to smoking | *S. p.*  *S. a.*  *M. c.*  *H. i.* | 127 (50.0)  32 (12.6)  152 (59.8)  88 (34.6) | 127 (50.0)  222 (87.4)  102 (40.2)  166 (65.4) |  | 26 (23.9)  30 (27.5)  36 (33.0)  20 (18.3) | 83 (76.1)  79 (72.5)  73 (67.0)  89 (81.7) |  | 2 (5.4)  18 (48.6)  5 (13.5)  0 (0.0) | 35 (94.6)  19 (51.4)  32 (86.5)  37 (100.0) |  |
| Total number of carriers and NCs | *S. p.*  *S. a.*  *M. c.*  *H. i.* | 162 (48.8)  38 (11.4)  199 (59.9)  113 (34.0) | 170 (51.2)  294 (88.6)  133 (40.1)  219 (66.0) |  | 40 (21.5)  56 (30.1)  70 (37.6)  37 (19.9) | 146 (78.5)  130 (69.9)  116 (62.4)  149 (80.1) |  | 4 (6.9)  29 (50.0)  9 (15.5)  0 (0.0) | 54 (93.1)  29 (50.0)  49 (84.5)  58 (100.0) |  |

*: Data of four children attending nursery were missing

NC, non-carrier

DCC, day-care centre
