# Supplementary figures and images for: Co-carriage of Staphylococcus aureus, Streptococcus pneumoniae, Haemophilus influenzae and Moraxella catarrhalis among three different age categories of children in Hungary
Source: PLoS One. 2020 Feb 7;15(2):e0229021. doi: 10.1371/journal.pone.0229021 (PMC7006921; doi:10.1371/journal.pone.0229021)

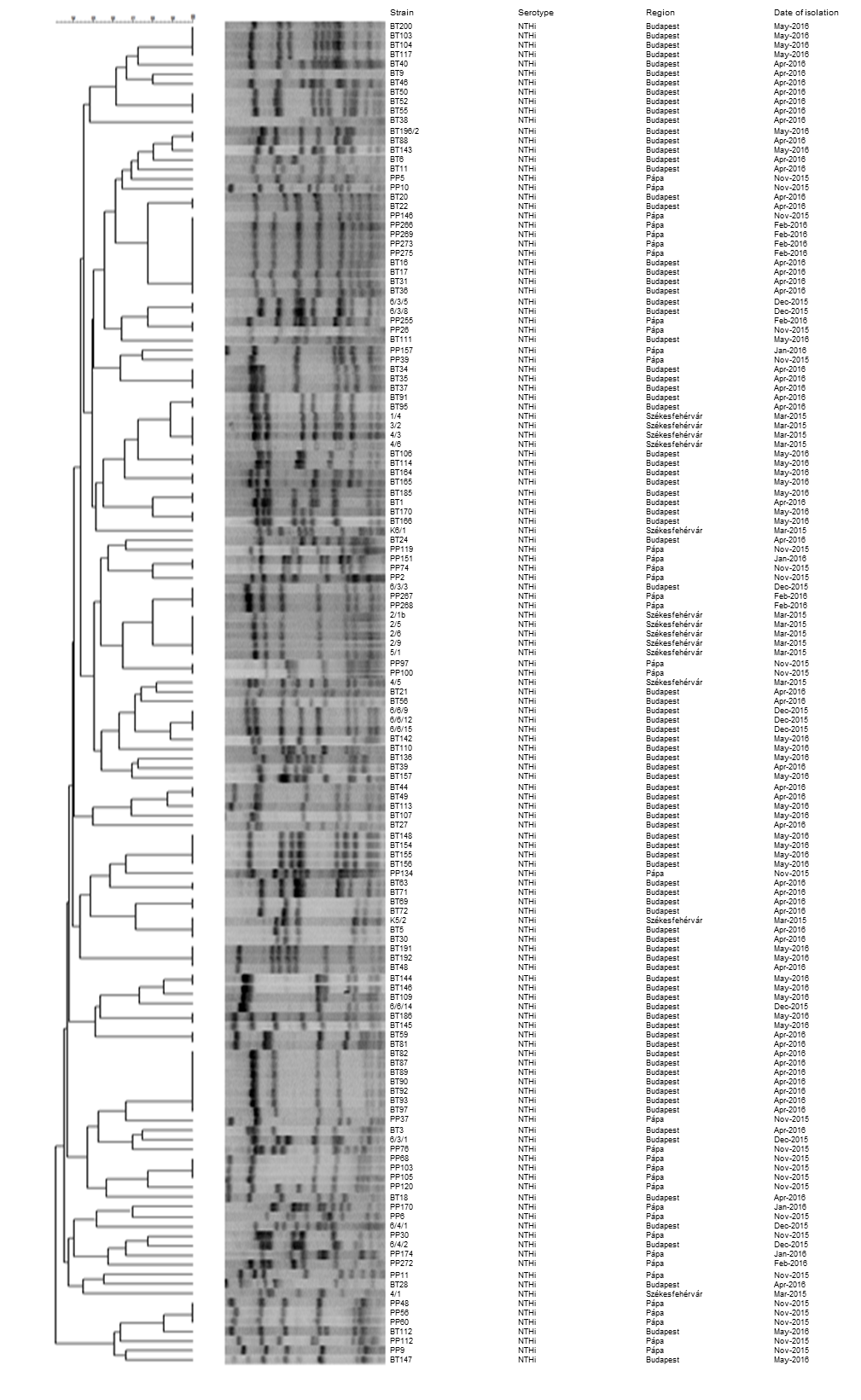

Supplement: S1 Fig — (DOCX) [file pone.0229021.s002.docx]

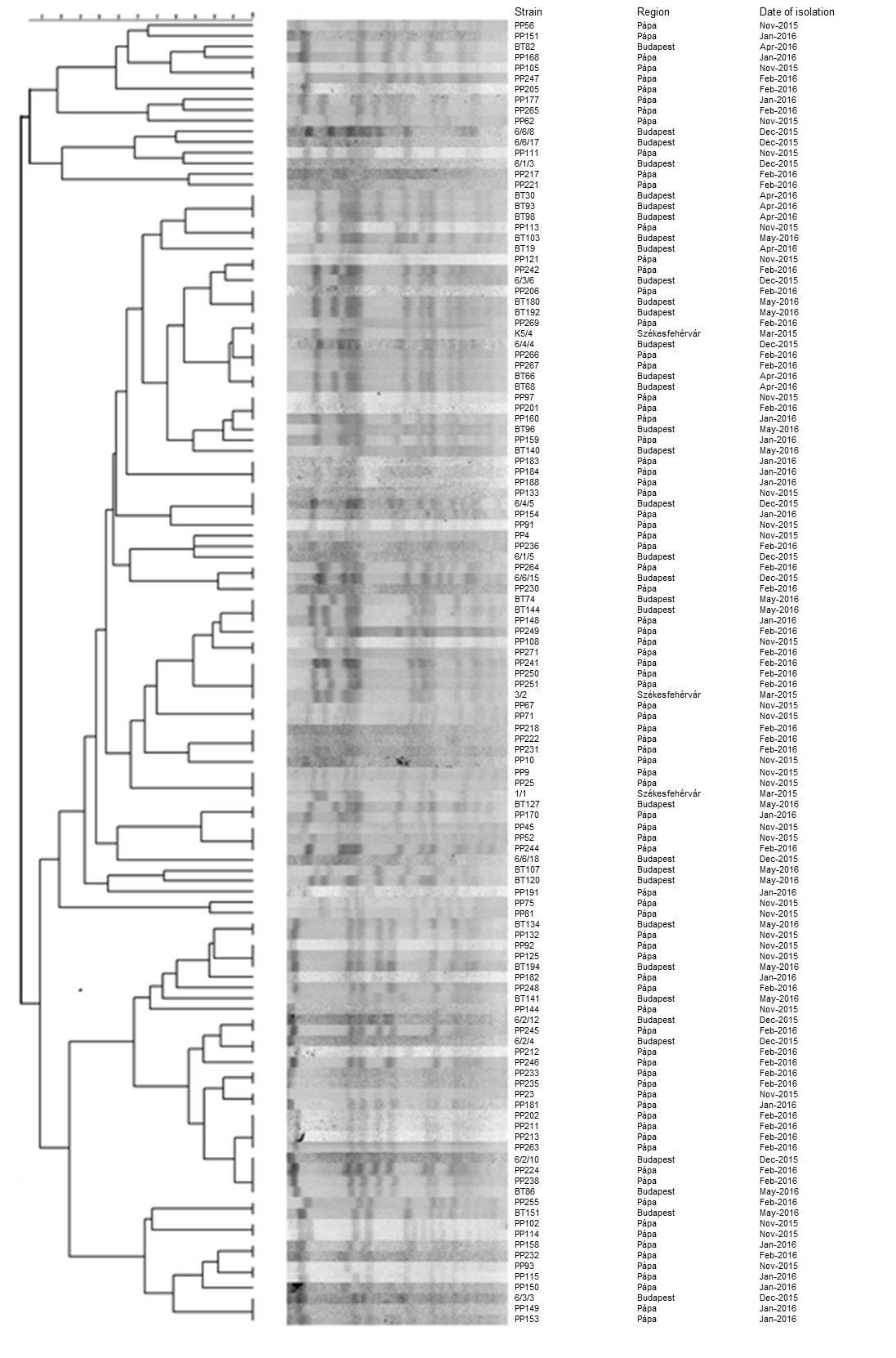

Supplement: S2 Fig — (DOCX) [file pone.0229021.s003.docx]
