# Supplementary material for: Co-carriage of Staphylococcus aureus, Streptococcus pneumoniae, Haemophilus influenzae and Moraxella catarrhalis among three different age categories of children in Hungary
Source: PLoS One. 2020 Feb 7;15(2):e0229021. doi: 10.1371/journal.pone.0229021 (PMC7006921; doi:10.1371/journal.pone.0229021)
Supplement: S1 Raw Images — (PDF) [file pone.0229021.s004.pdf]

Fig 4 and S1 Fig were generated from these original *H. influenzae* PFGE pictures.

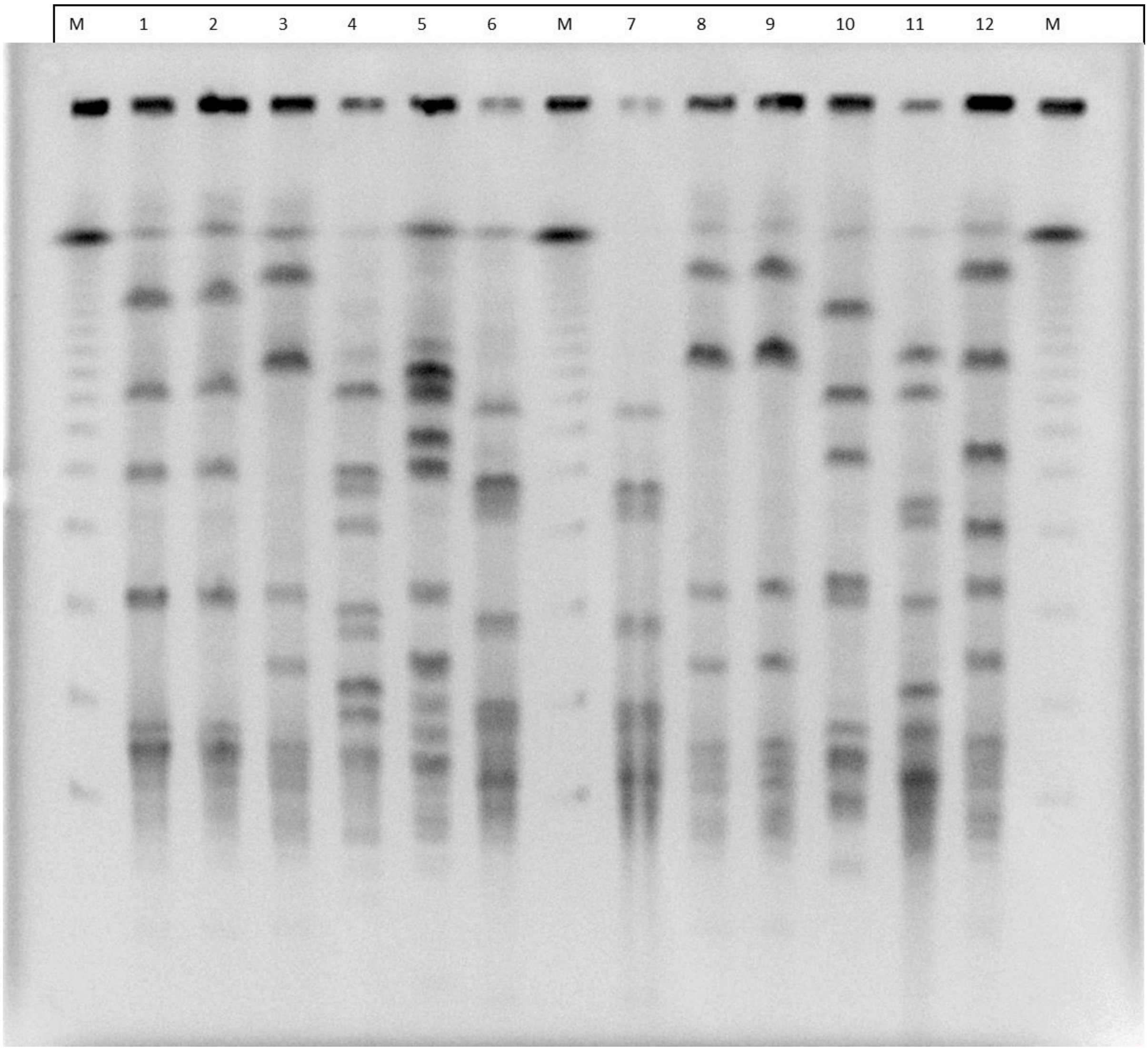

Lanes: M: molecular weight marker - lambda (λ) ladder (CHEF DNA size standard; catalog no. 170-3635; Bio-Rad) 1. PP56 2. PP60 3. PP68 4. PP74 5. PP76 6. PP97 7. PP100 8. PP103 9. PP105 10. PP112 11. PP119 12. PP120

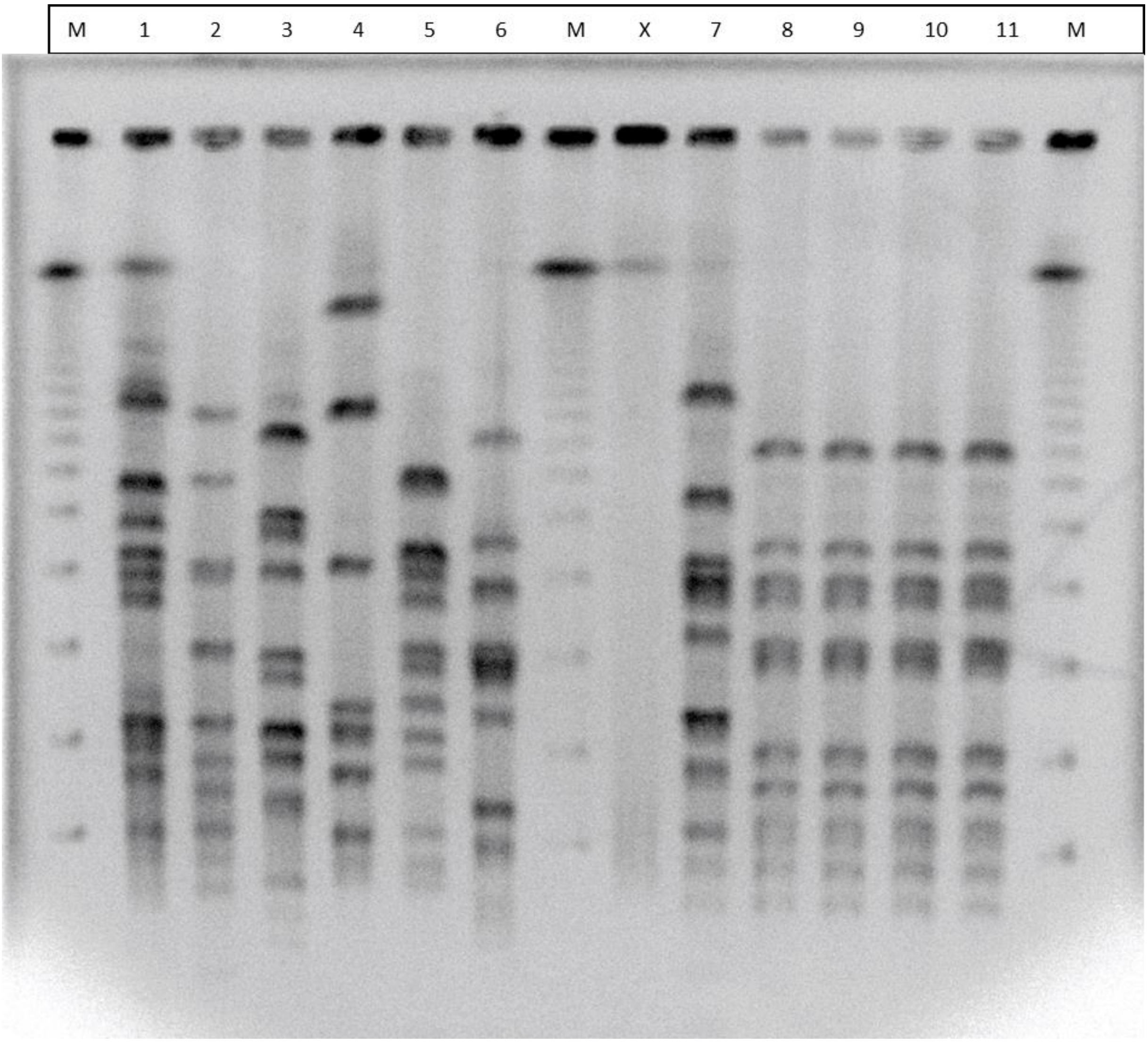

Lanes: M: molecular weight marker - lambda (λ) ladder (CHEF DNA size standard; catalog no. 170-3635; Bio-Rad) 1. PP134 2. PP146 3. PP151 4. PP157 5. PP170 6. PP174 7. PP255 8. PP257 9. PP259 10. PP263 11. PP264

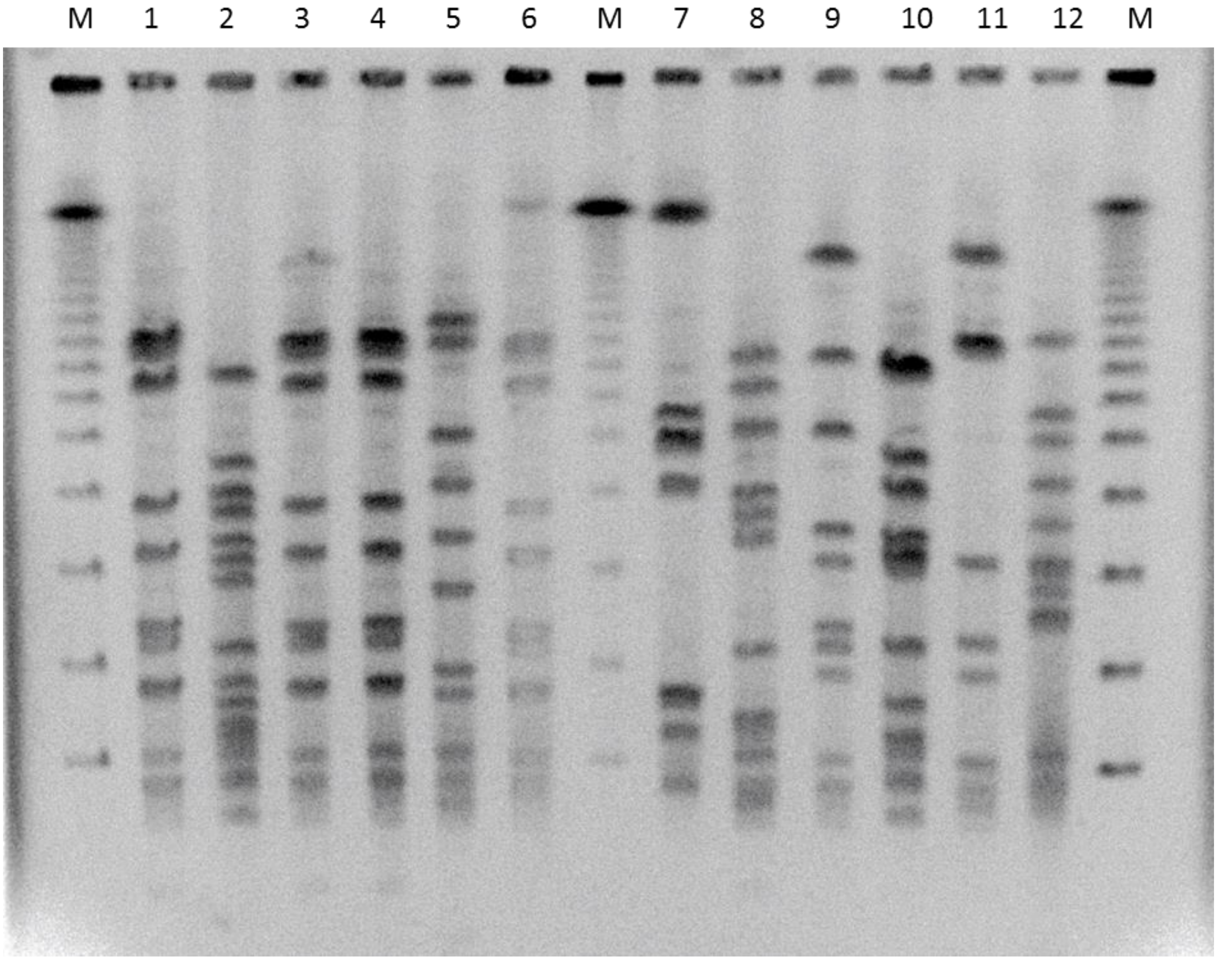

Lanes: M: molecular weight marker - lambda (λ) ladder (CHEF DNA size standard; catalog no. 170-3635; Bio-Rad) 1. 1/4 2. 2/8 3. 3/2 4. 4/3 5. 4/5 6. 4/6 7. K5/2 8. K6/1 9. PP10 10. PP17 11. PP37 12. 6/4/1

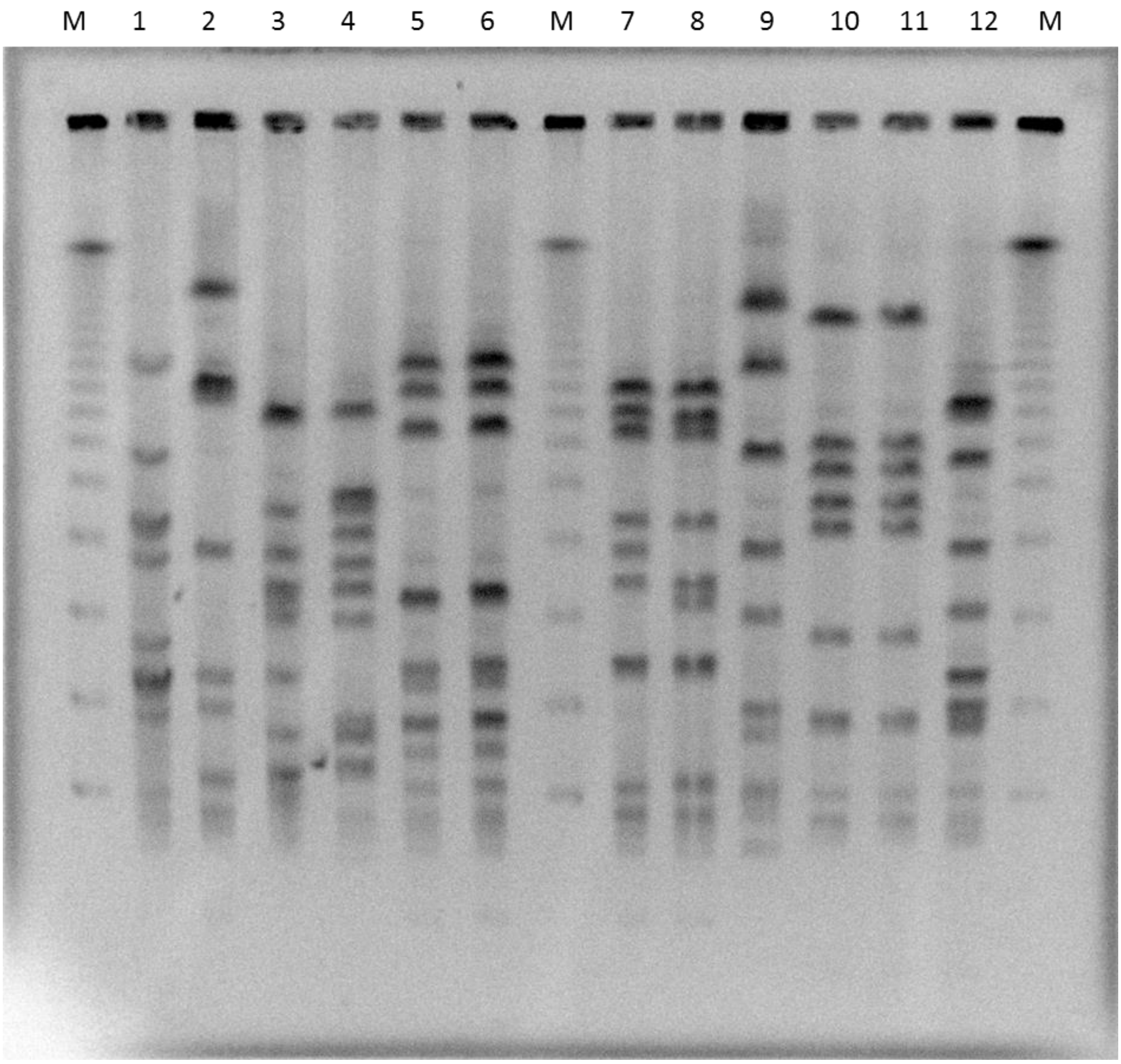

Lanes: M: molecular weight marker - lambda (λ) ladder (CHEF DNA size standard; catalog no. 170-3635; Bio-Rad) 1. BT111 2. BT113 3. BT115 4. BT136 5. BT164 6. BT165 7. BT170 8. BT185 9. BT186 10. BT191 11. BT192 12. BT196/2

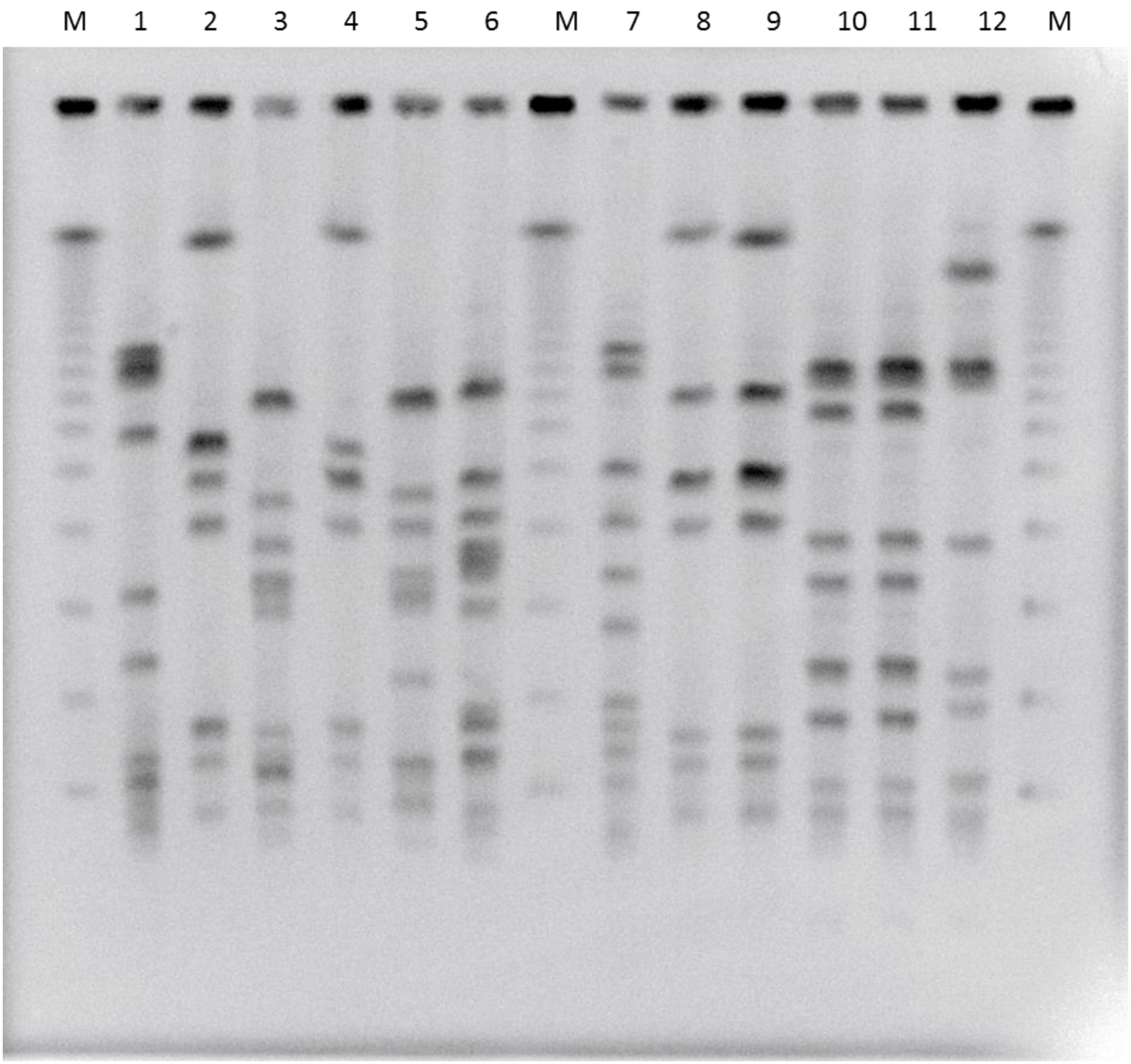

Lanes: M: molecular weight marker - lambda (λ) ladder (CHEF DNA size standard; catalog no. 170-3635; Bio-Rad) 1. BT3 2. BT5 3. BT25 4. BT30 5. BT32 6. BT39 7. BT56 8. BT69 9. BT72 10. BT91 11. BT95 12. BT107

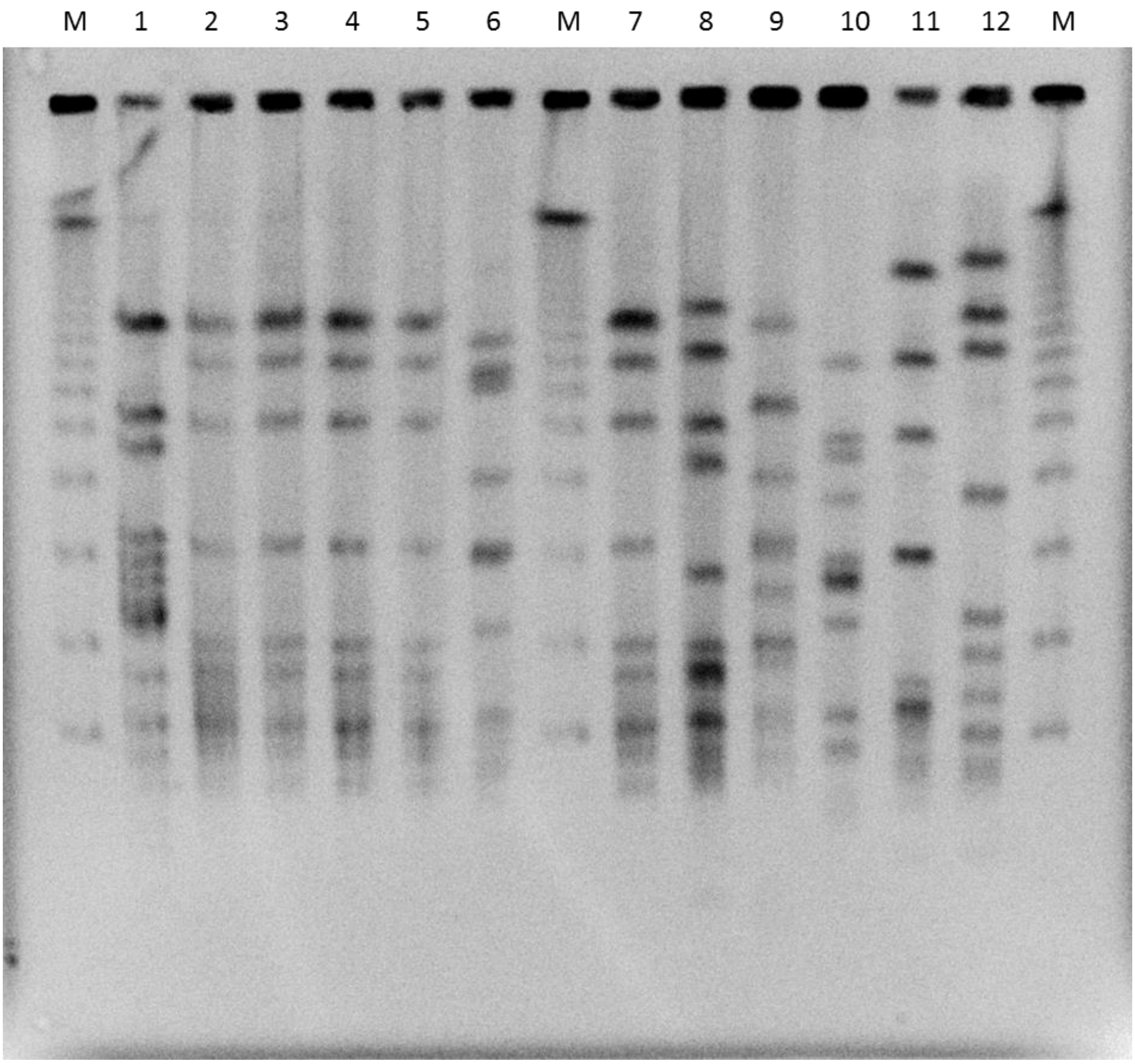

Lanes: M: molecular weight marker - lambda (λ) ladder (CHEF DNA size standard; catalog no. 170-3635; Bio-Rad) 1. BT200 2. 2/1B 3. 2/5 4. 2/6 5. 2/9 6. 4/1 7. 5/1 8. PP2 9. PP5 10. PP6 11. PP9 12. PP11

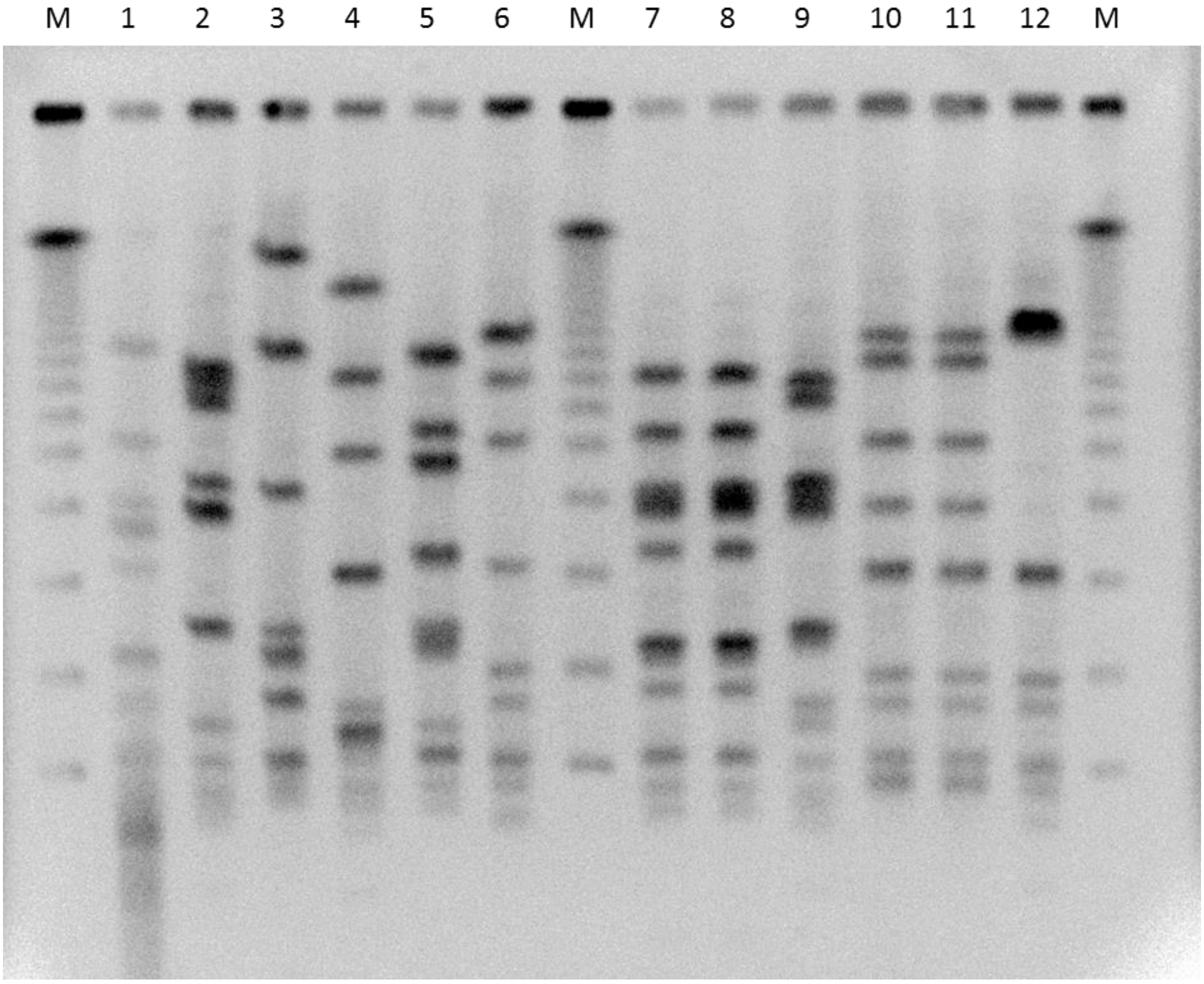

Lanes: M: molecular weight marker - lambda (λ) ladder (CHEF DNA size standard; catalog no. 170-3635; Bio-Rad) 1. PP26 2. PP30 3. PP39 4. PP48 5. 6/3/1 6. 6/3/3 7. 6/3/5 8. 6/3/8 9. 6/4/2 10. 6/6/9 11. 6/6/12 12. 6/6/14

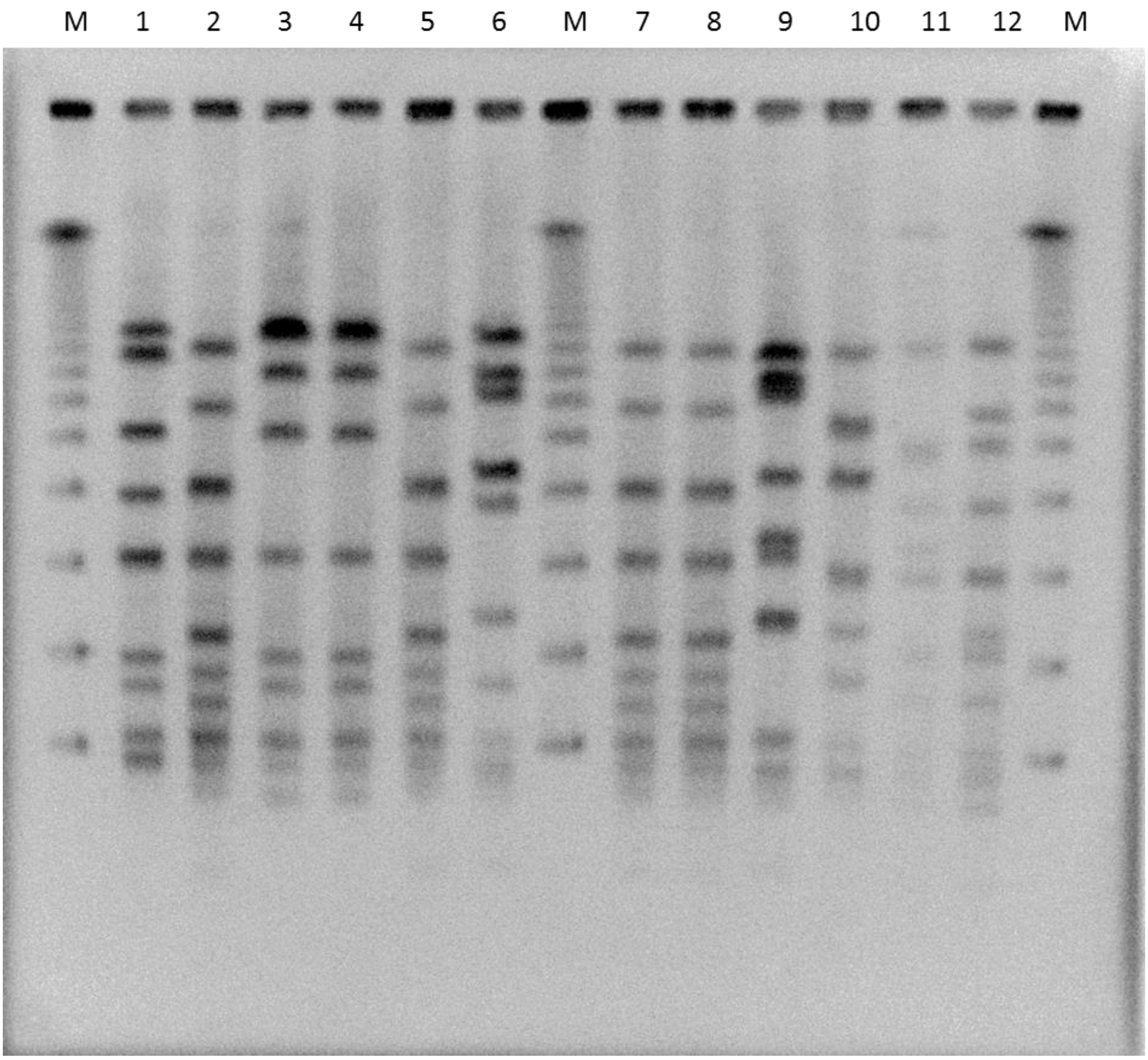

Lanes: M: molecular weight marker - lambda (λ) ladder (CHEF DNA size standard; catalog no. 170-3635; Bio-Rad) 1. 6/6/15 2. PP266 3. PP267 4. PP268 5. PP269 6. PP272 7. PP273 8. PP275 9. BT1 10. BT6 11. BT9 12. BT11

M X X 1 2 3 4 M 5 6 7 8 9 10 M

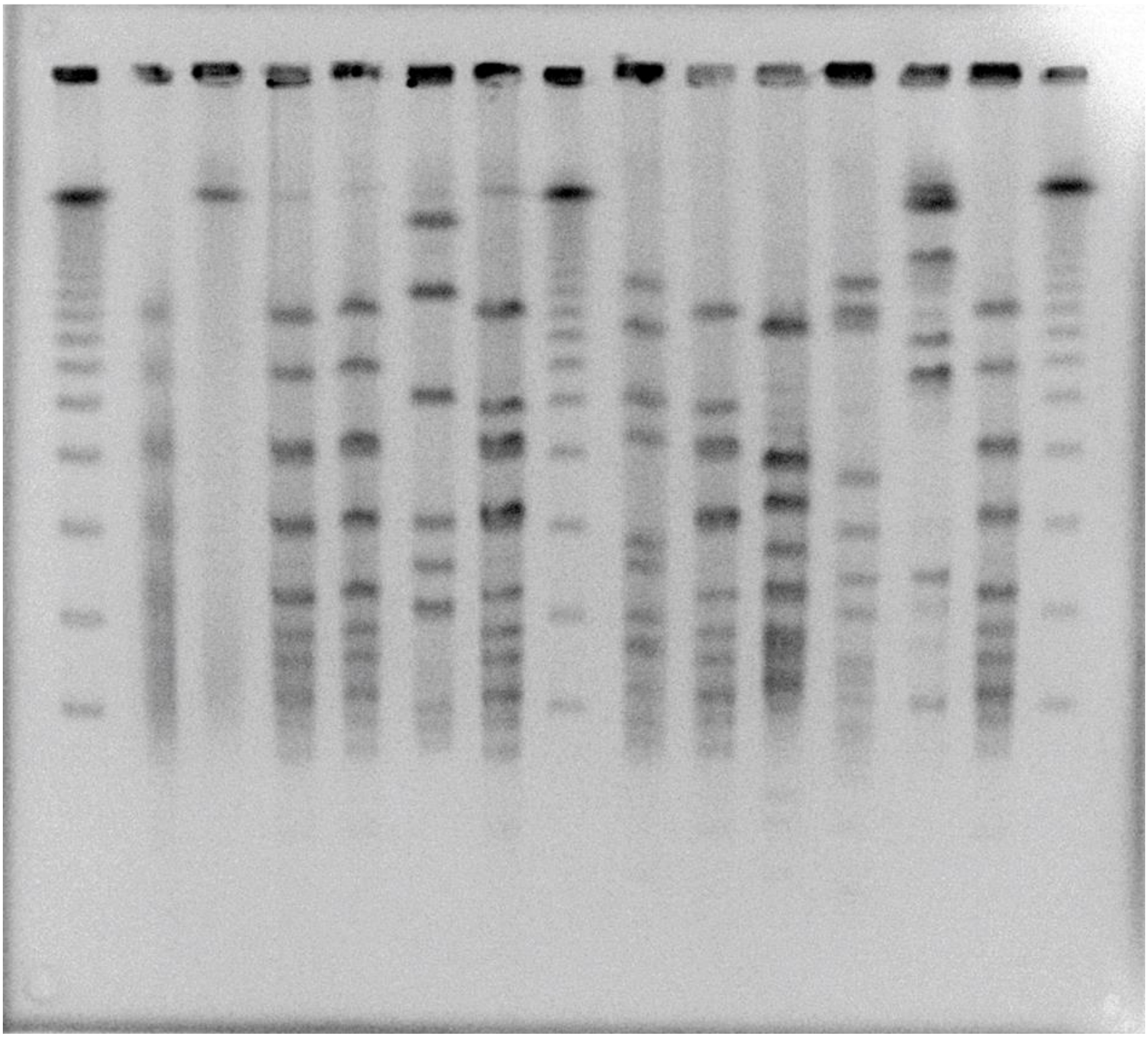

Lanes: M: molecular weight marker - lambda (λ) ladder (CHEF DNA size standard; catalog no. 170-3635; Bio-Rad) 1. BT16 2. BT17 3. BT18 4. BT20 5. BT21 6. BT22 7. BT24 8. BT27 9. BT28 10. BT31

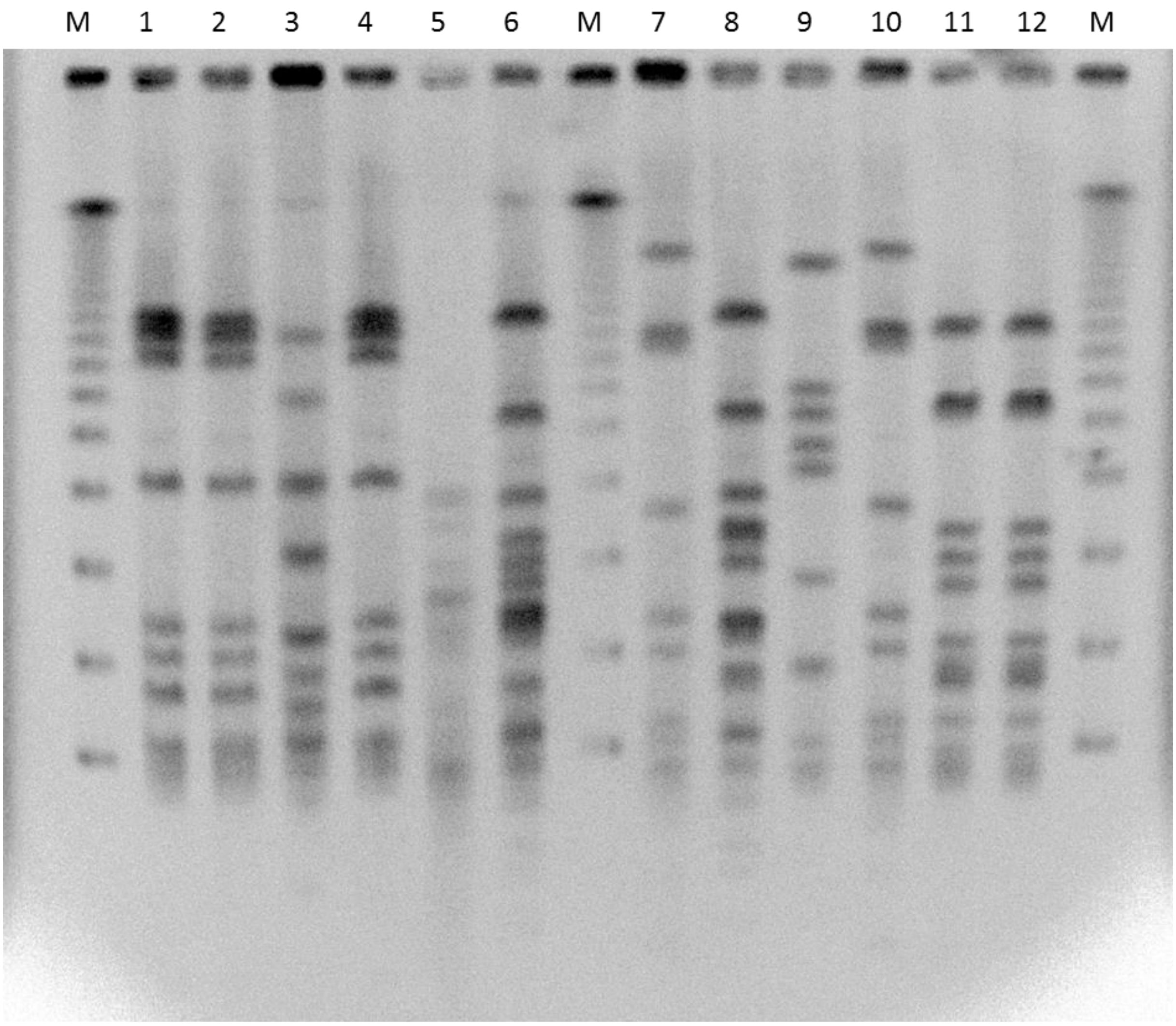

Lanes: M: molecular weight marker - lambda (λ) ladder (CHEF DNA size standard; catalog no. 170-3635; Bio-Rad) 1. BT34 2. BT35 3. BT36 4. BT37 5. BT38 6. BT40 7. BT44 8. BT46 9. BT48 10. BT49 11. BT50 12. BT52

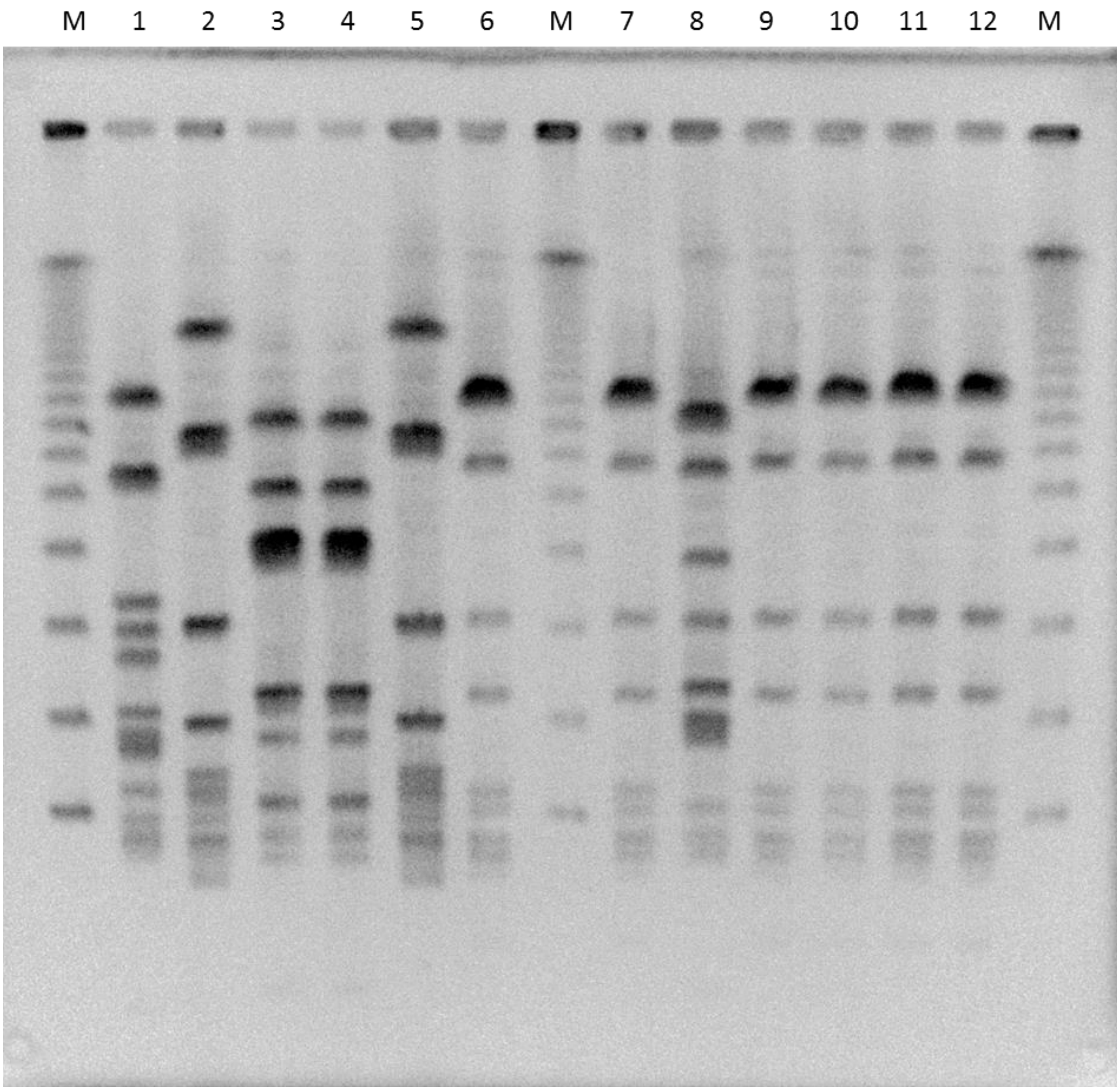

Lanes: M: molecular weight marker - lambda (λ) ladder (CHEF DNA size standard; catalog no. 170-3635; Bio-Rad) 1. BT55 2. BT59 3. BT63 4. BT71 5. BT81 6. BT82 7. BT87 8. BT88 9. BT89 10. BT90 11. BT92 12. BT93

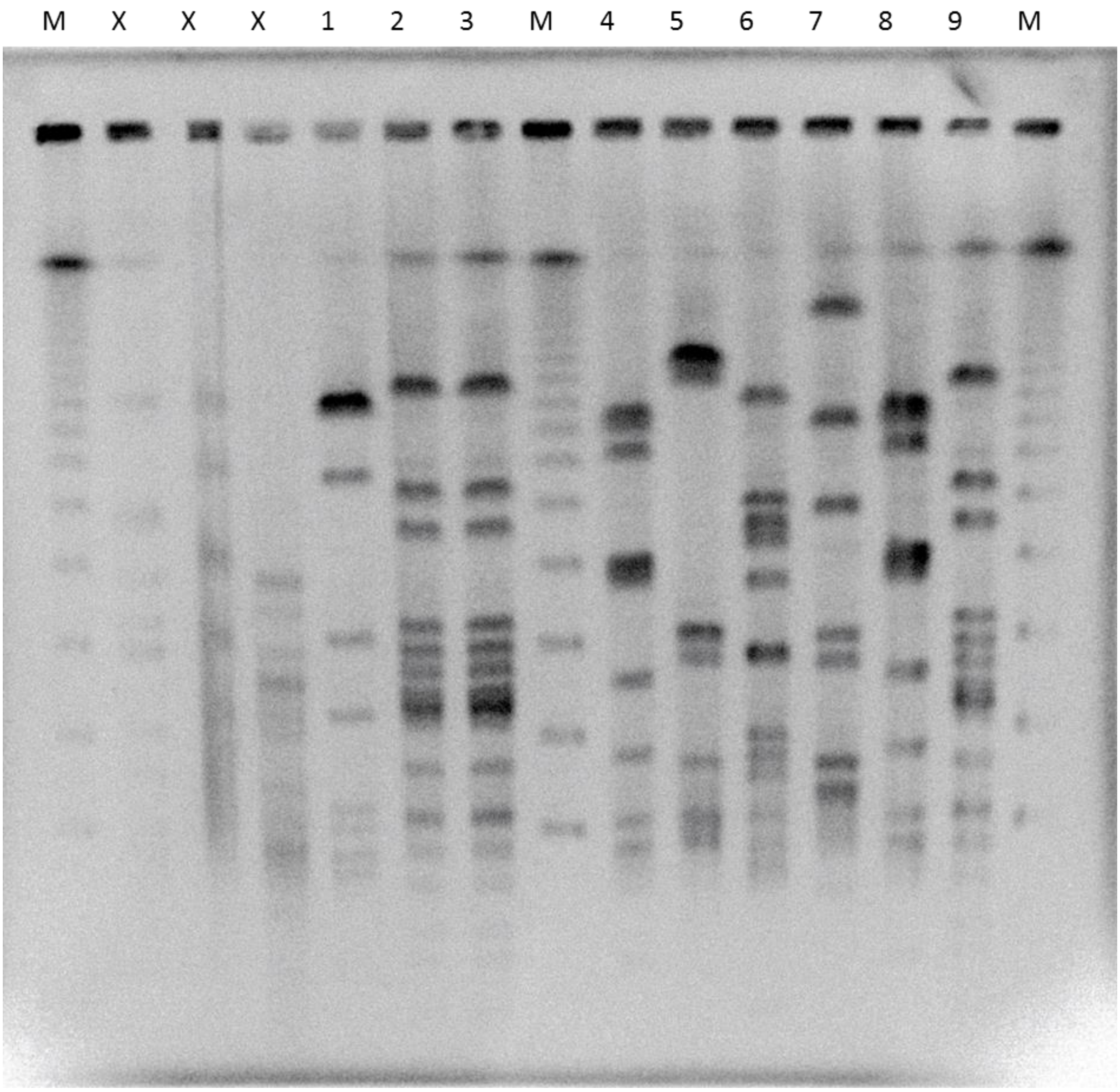

Lanes: M: molecular weight marker - lambda (λ) ladder (CHEF DNA size standard; catalog no. 170-3635; Bio-Rad) 1. BT97 2. BT103 3. BT104 4. BT106 5. BT109 6. BT110 7. BT112 8. BT114 9. BT117

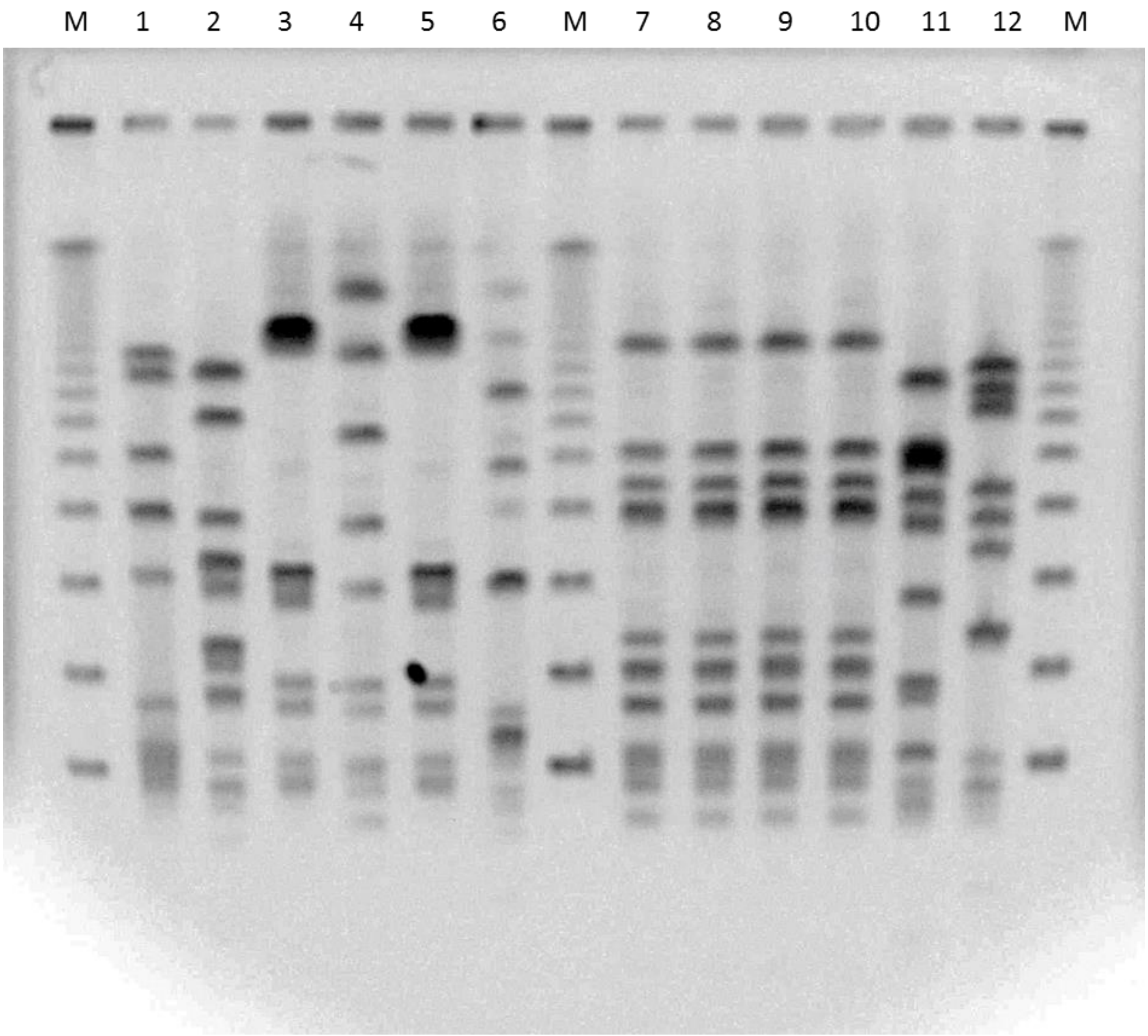

Lanes: M: molecular weight marker - lambda (λ) ladder (CHEF DNA size standard; catalog no. 170-3635; Bio-Rad) 1. BT142 2. BT143 3. BT144 4. BT145 5. BT146 6. BT147 7. BT148 8. BT154 9. BT155 10. BT156 11. BT157 12. BT166
