# Supplementary material for: Co-carriage of Staphylococcus aureus, Streptococcus pneumoniae, Haemophilus influenzae and Moraxella catarrhalis among three different age categories of children in Hungary
Source: PLoS One. 2020 Feb 7;15(2):e0229021. doi: 10.1371/journal.pone.0229021 (PMC7006921; doi:10.1371/journal.pone.0229021)
Supplement: S2 Raw Images — (PDF) [file pone.0229021.s005.pdf]

Fig 5 and S2 Fig were generated from these original *S. aureus* PFGE pictures.

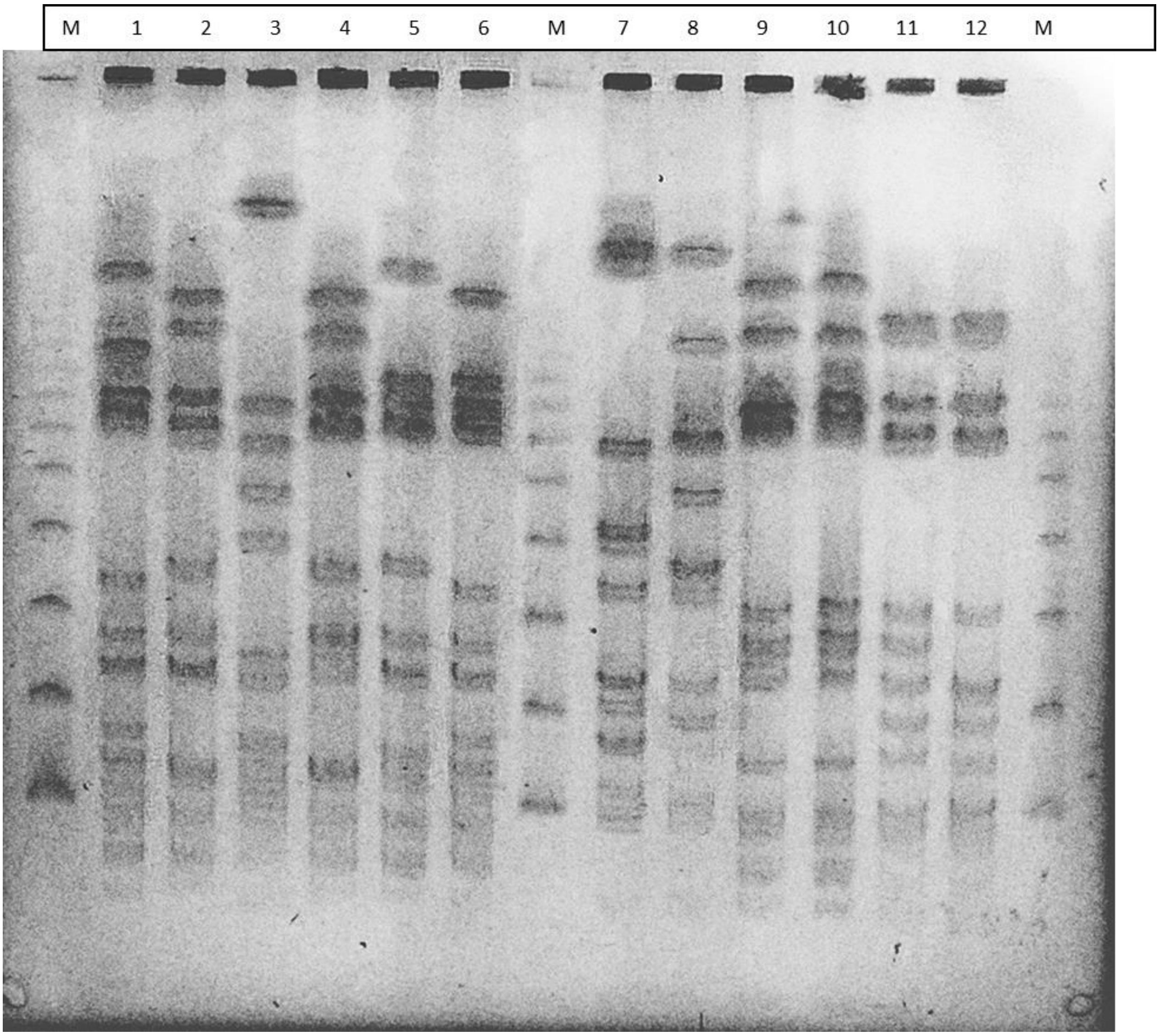

Lanes: M: molecular weight marker - lambda (λ) ladder (CHEF DNA size standard; catalog no. 170-3635; Bio-Rad) 1. PP4 2. PP9 3. PP23 4. PP25 5. PP45 6. PP52 7. PP56 8. PP62 9. PP67 10. PP71 11. PP75 12. PP81

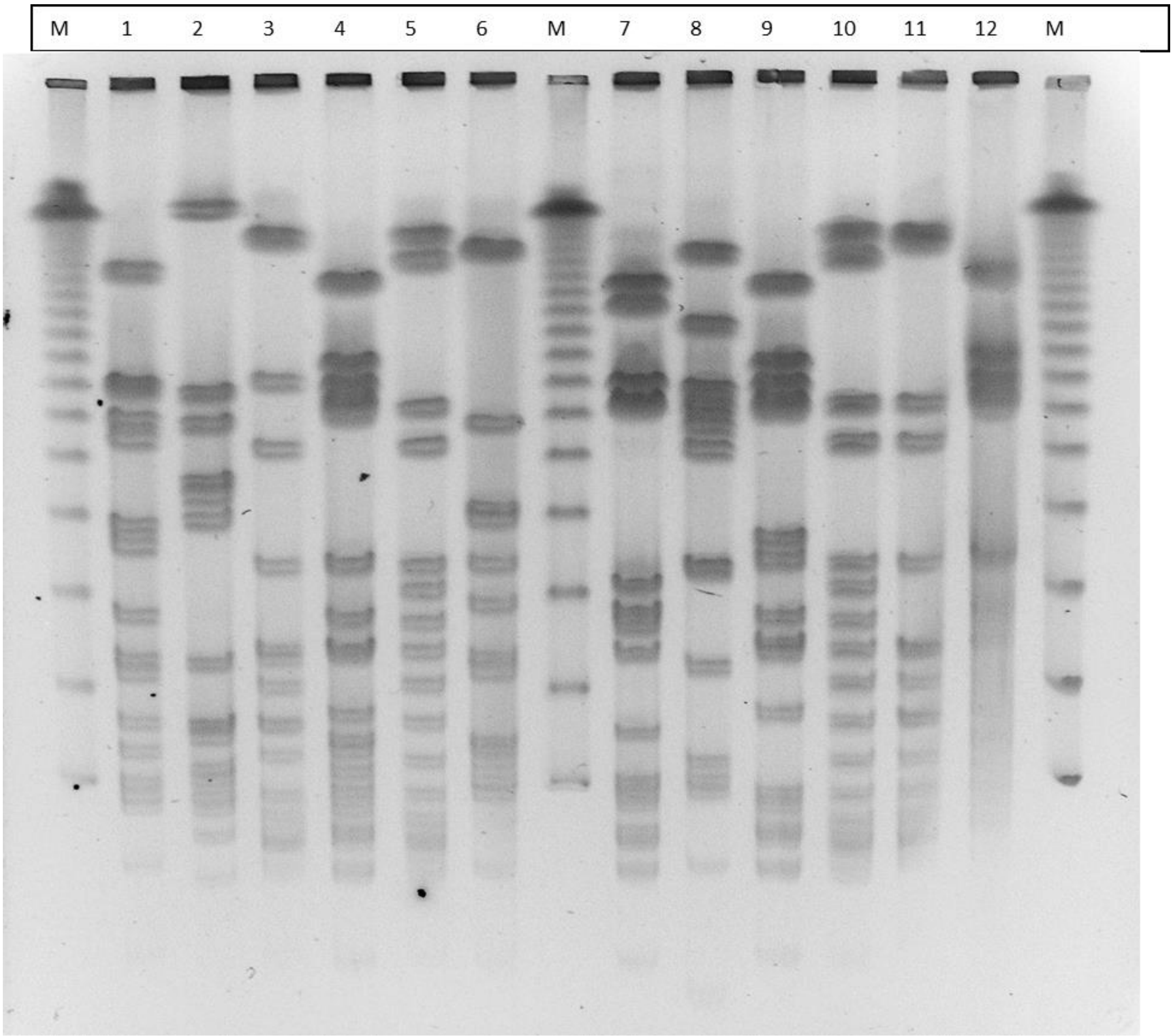

Lanes: M: molecular weight marker - lambda (λ) ladder (CHEF DNA size standard; catalog no. 170-3635; Bio-Rad) 1. PP91 2. PP92 3. PP93 4. PP97 5. PP102 6. PP105 7. PP108 8. PP111 9. PP113 10. PP114 11. PP115 12. PP121

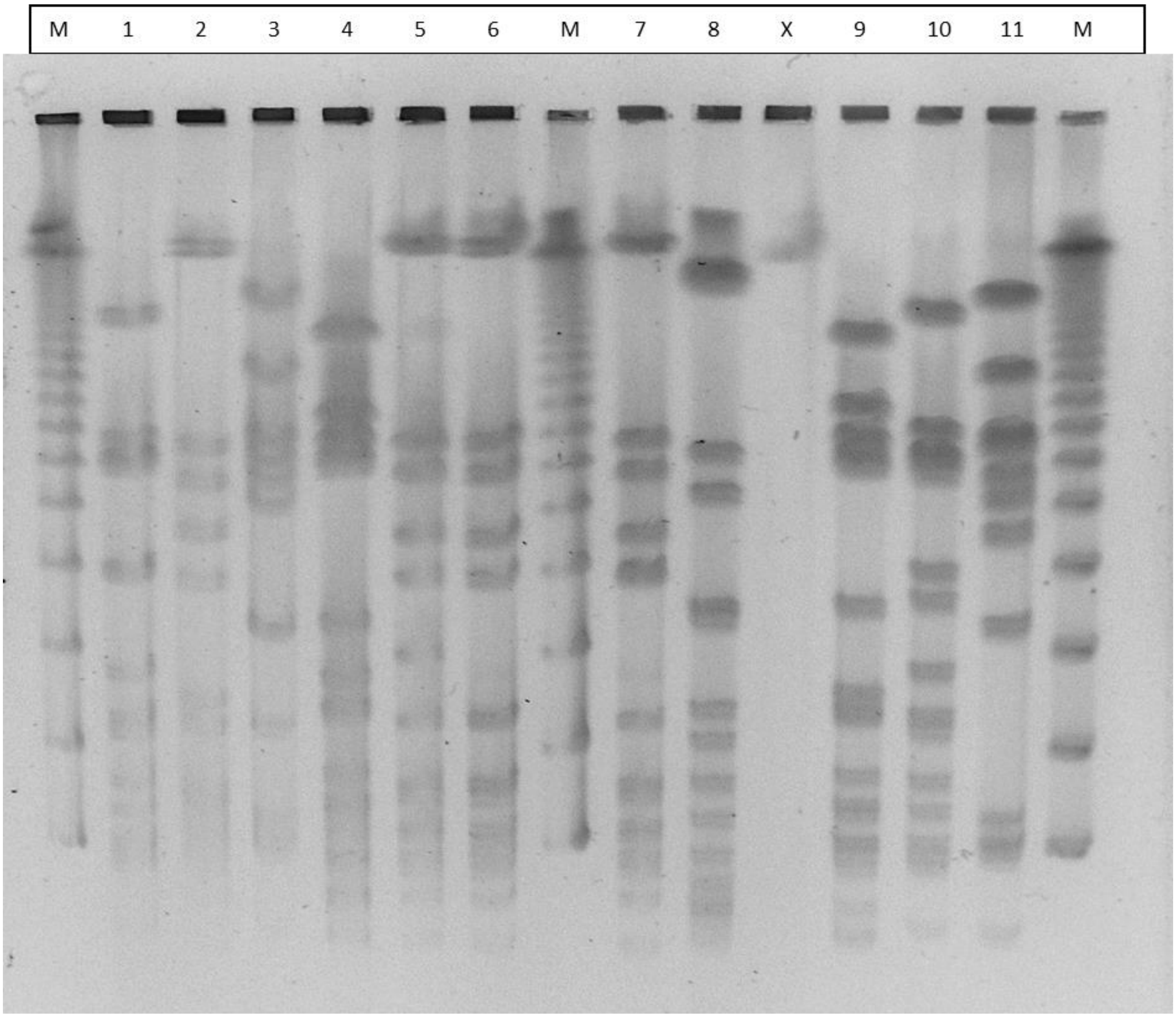

Lanes: M: molecular weight marker - lambda (λ) ladder (CHEF DNA size standard; catalog no. 170-3635; Bio-Rad) 1. PP133 2. PP134 3. 6/1/3 4. 6/1/5 5. 6/2/4 6. 6/2/10 7. 6/2/12 8. 6/3/3 9. 6/4/4 10. 6/4/5 11. 6/6/8

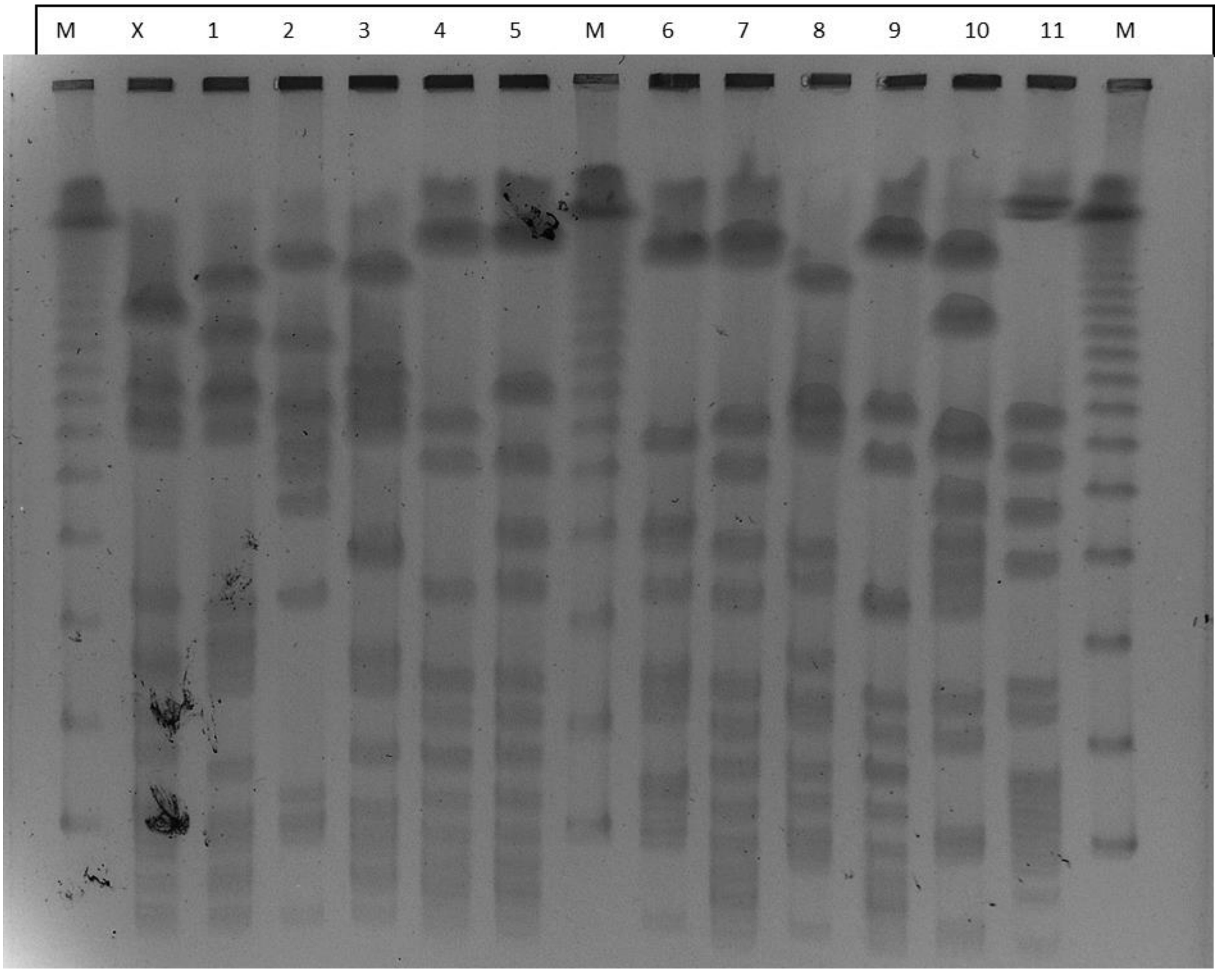

Lanes: M: molecular weight marker - lambda (λ) ladder (CHEF DNA size standard; catalog no. 170-3635; Bio-Rad) 1. PP10 2. 6/6/17 3. 6/6/18 4. PP149 5. PP150 6. PP151 7. PP153 8. PP154 9. PP158 10. PP177 11. PP181

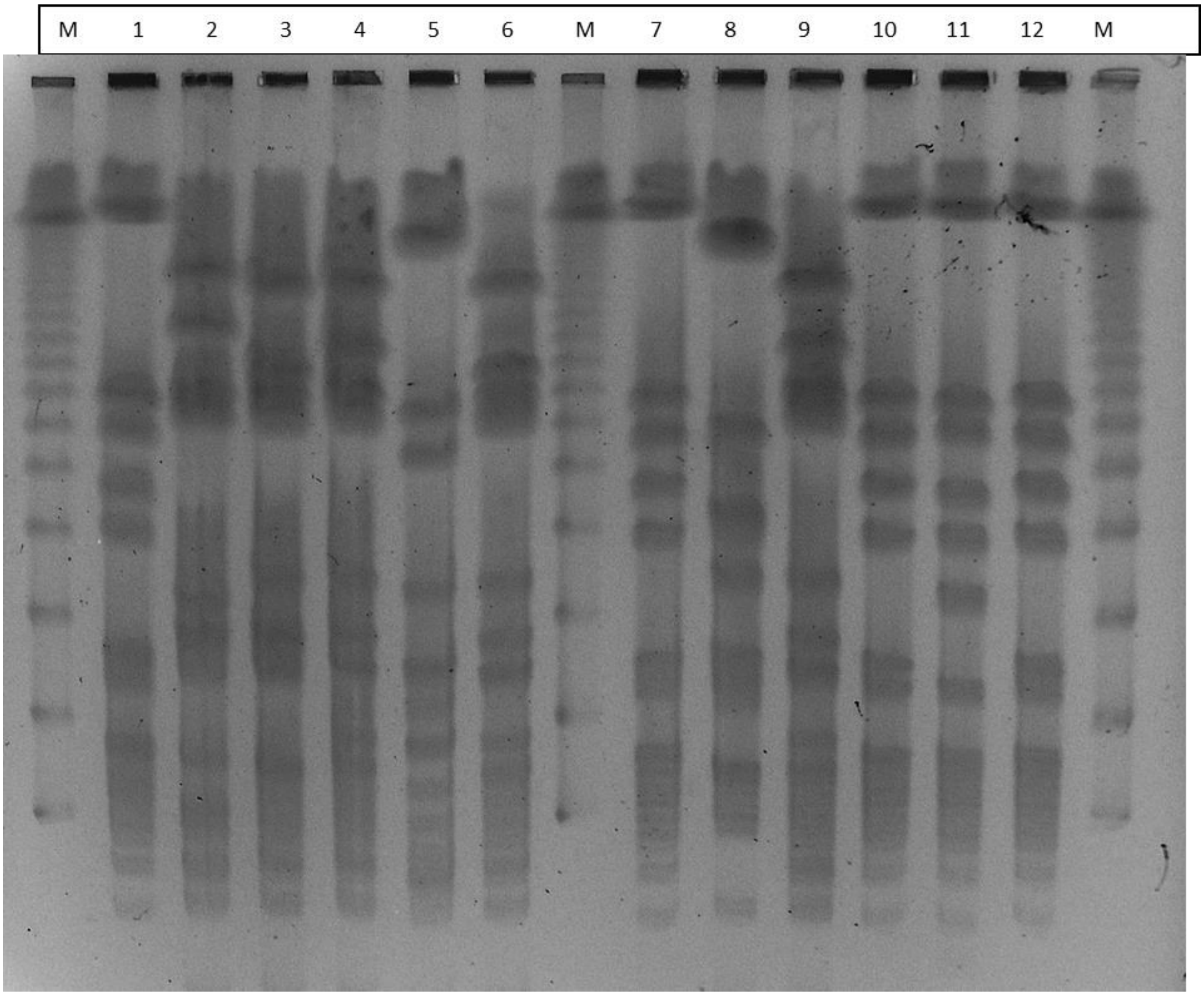

Lanes: M: molecular weight marker - lambda (λ) ladder (CHEF DNA size standard; catalog no. 170-3635; Bio-Rad) 1. PP182 2. PP183 3. PP184 4. PP188 5. PP191 6. PP201 7. PP202 8. PP205 9. PP206 10. PP211 11. PP212 12. PP213

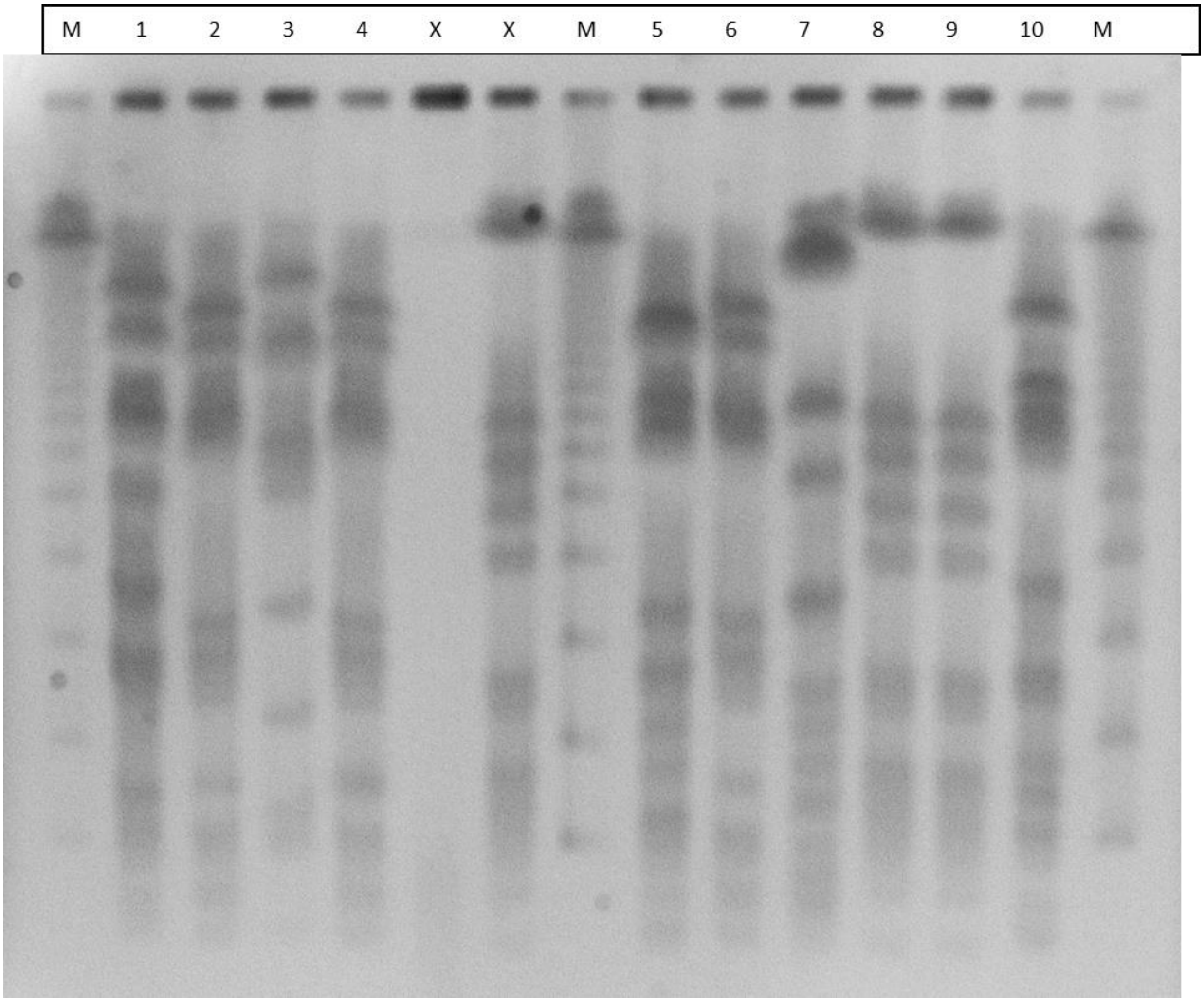

Lanes: M: molecular weight marker - lambda (λ) ladder (CHEF DNA size standard; catalog no. 170-3635; Bio-Rad) 1. PP217 2. PP218 3. PP221 4. PP222 5. PP230 6. PP231 7. PP232 8. PP233 9. PP235 10. PP236

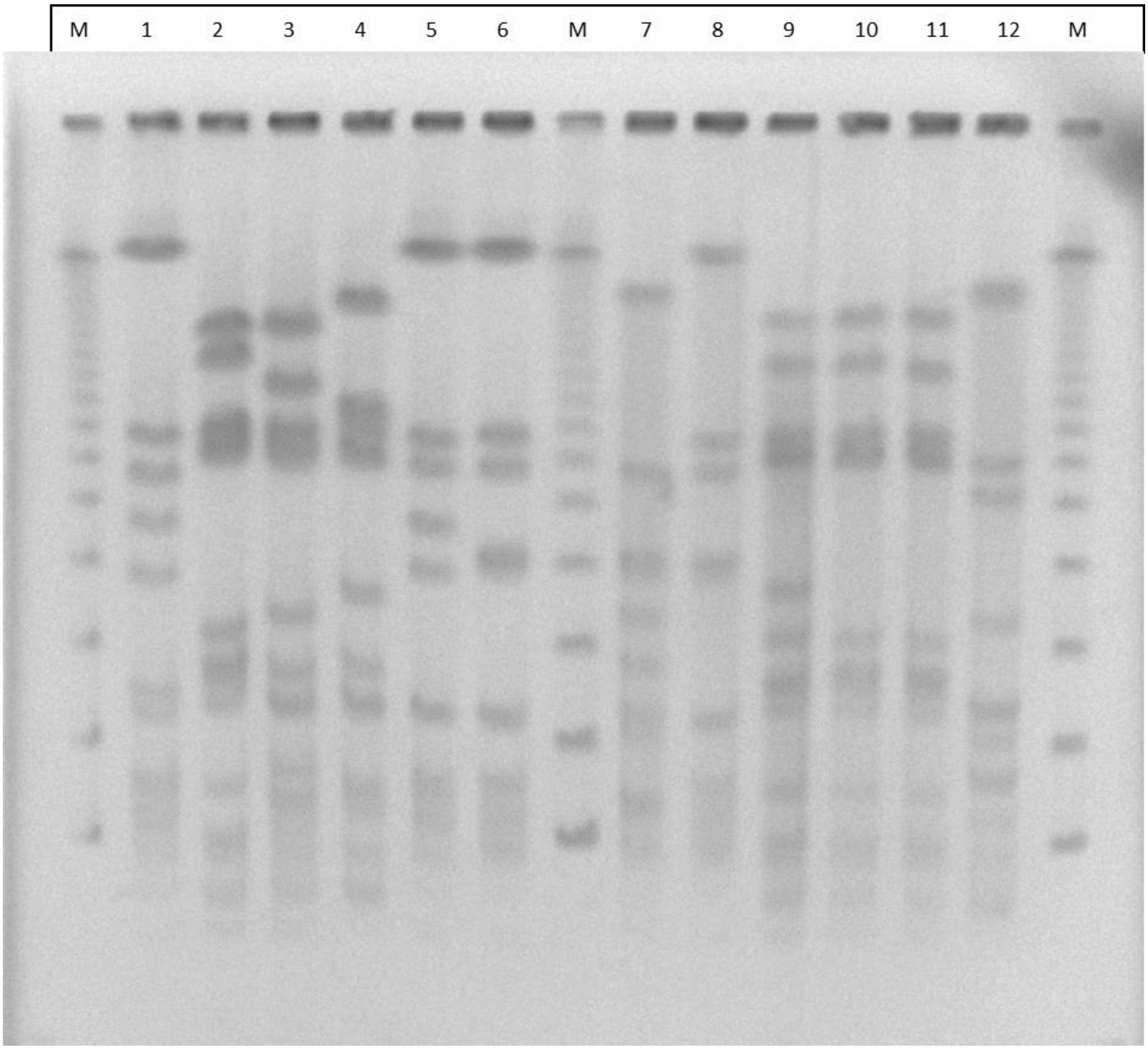

Lanes: M: molecular weight marker - lambda (λ) ladder (CHEF DNA size standard; catalog no. 170-3635; Bio-Rad) 1. PP238 2. PP241 3. PP242 4. PP244 5. PP245 6. PP246 7. PP247 8. PP248 9. PP249 10. PP250 11. PP251 12. PP255

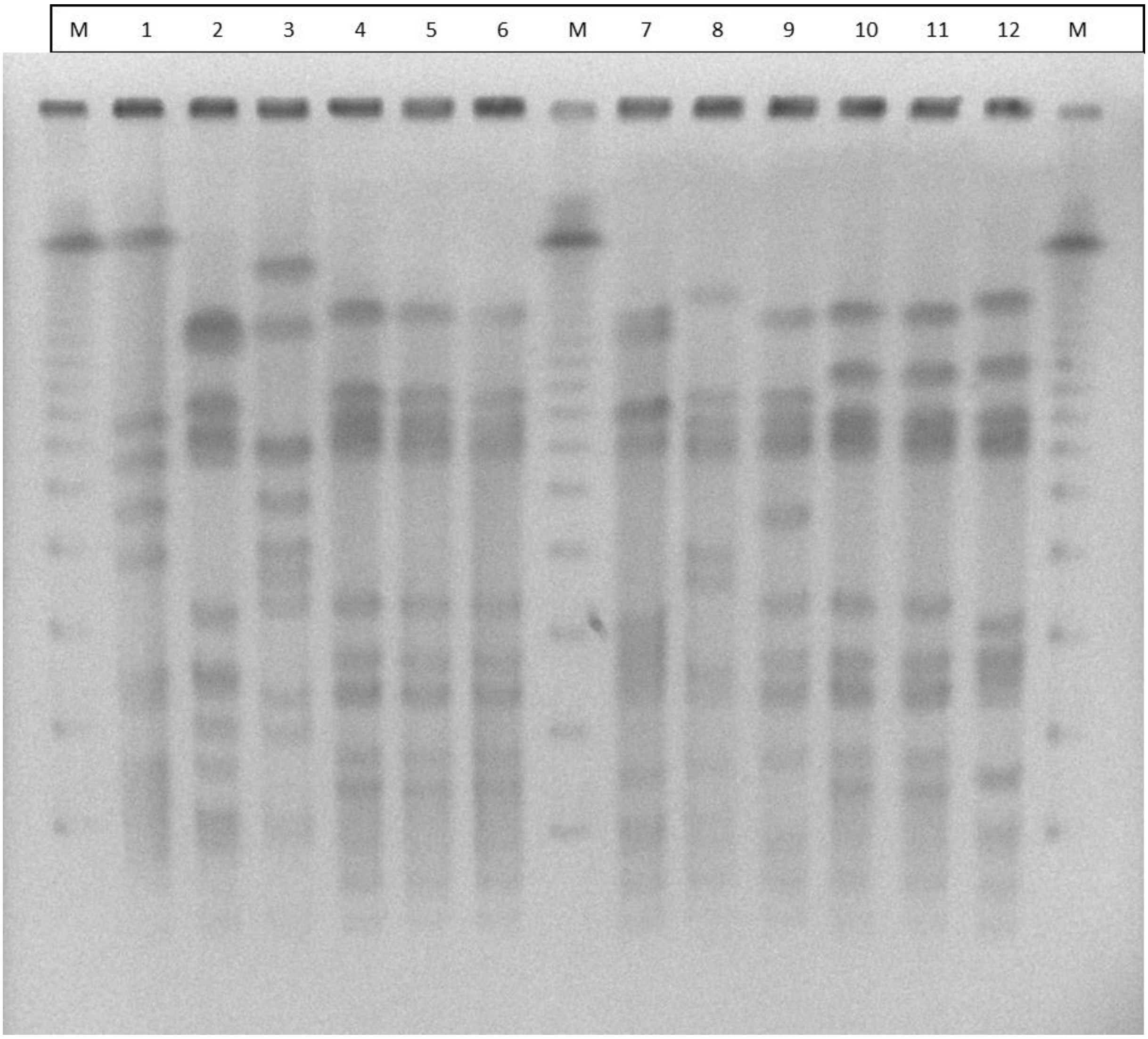

Lanes: M: molecular weight marker - lambda (λ) ladder (CHEF DNA size standard; catalog no. 170-3635; Bio-Rad) 1. PP263 2. PP264 3. PP265 4. PP266 5. PP267 6. PP269 7. PP271 8. BT19 9. BT30 10. BT66 11. BT68 12. BT74

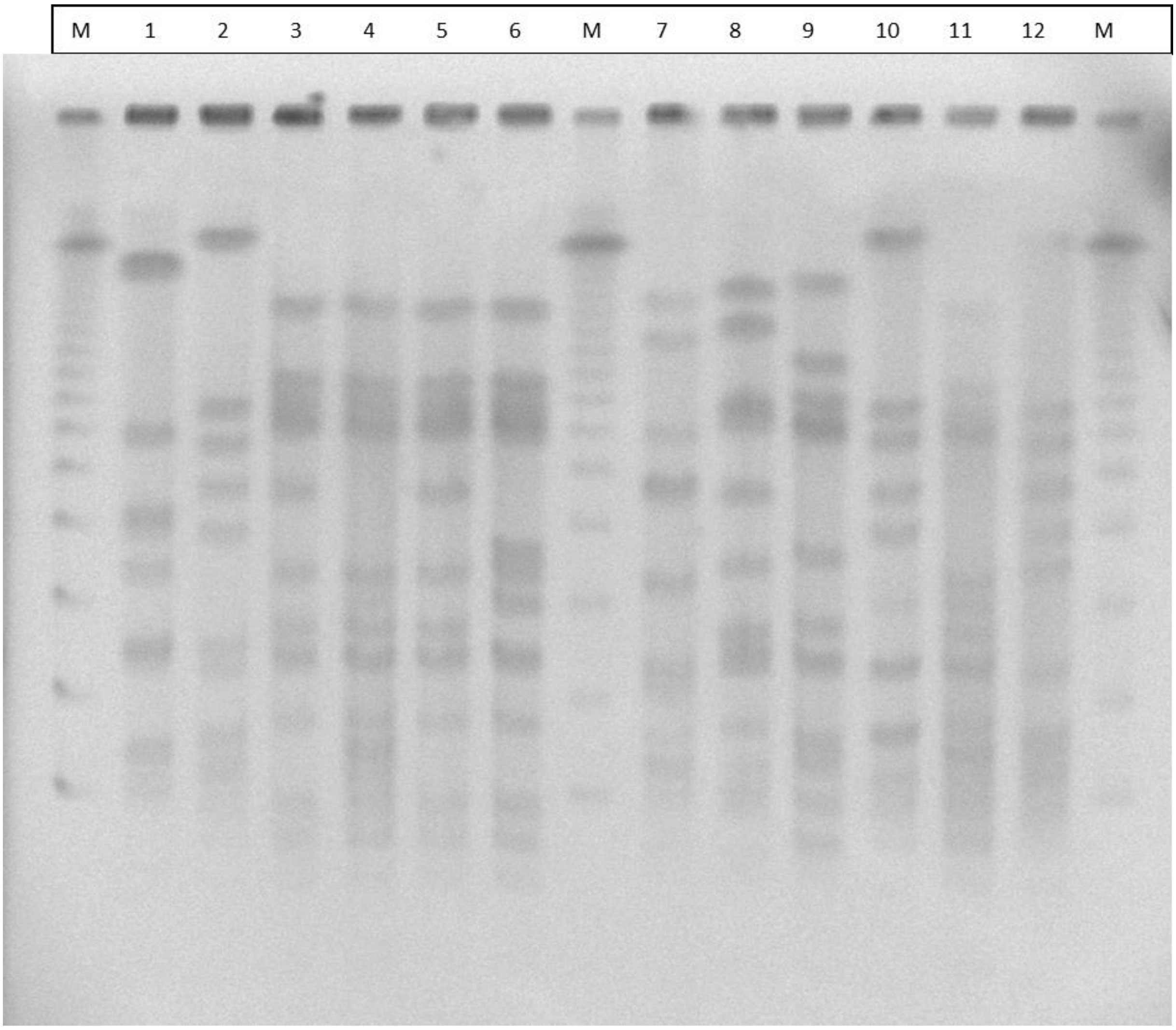

Lanes: M: molecular weight marker - lambda (λ) ladder (CHEF DNA size standard; catalog no. 170-3635; Bio-Rad) 1. BT82 2. BT86 3. BT93 4. BT96 5. BT98 6. BT103 7. BT107 8. BT120 9. BT127 10. BT134 11. BT140 12. BT141

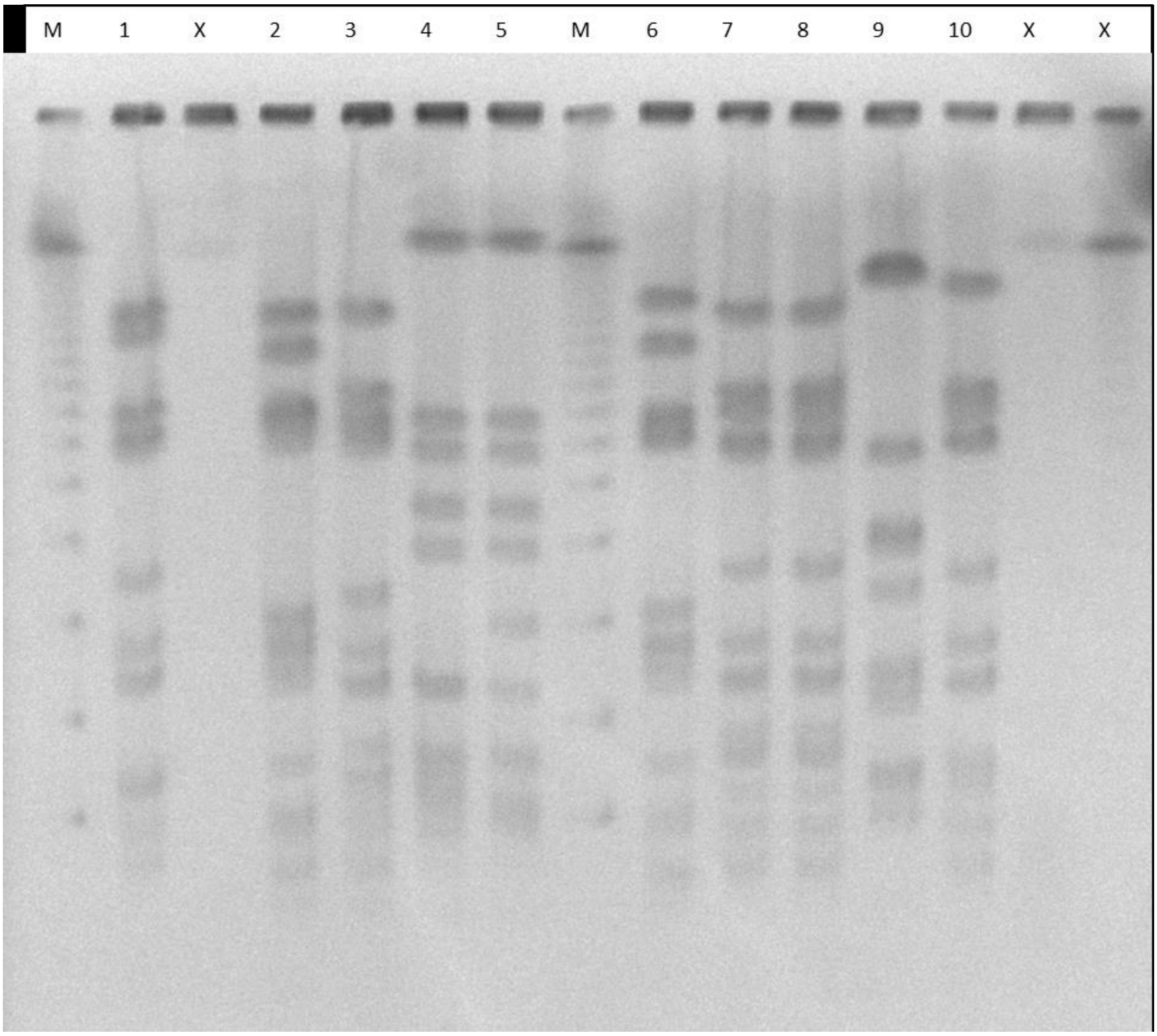

Lanes: M: molecular weight marker - lambda (λ) ladder (CHEF DNA size standard; catalog no. 170-3635; Bio-Rad) 1. 1/1 2. 3/2 3. K5/4 4. PP125 5. PP132 6. PP148 7. PP159 8. PP160 9. PP168 10. PP170

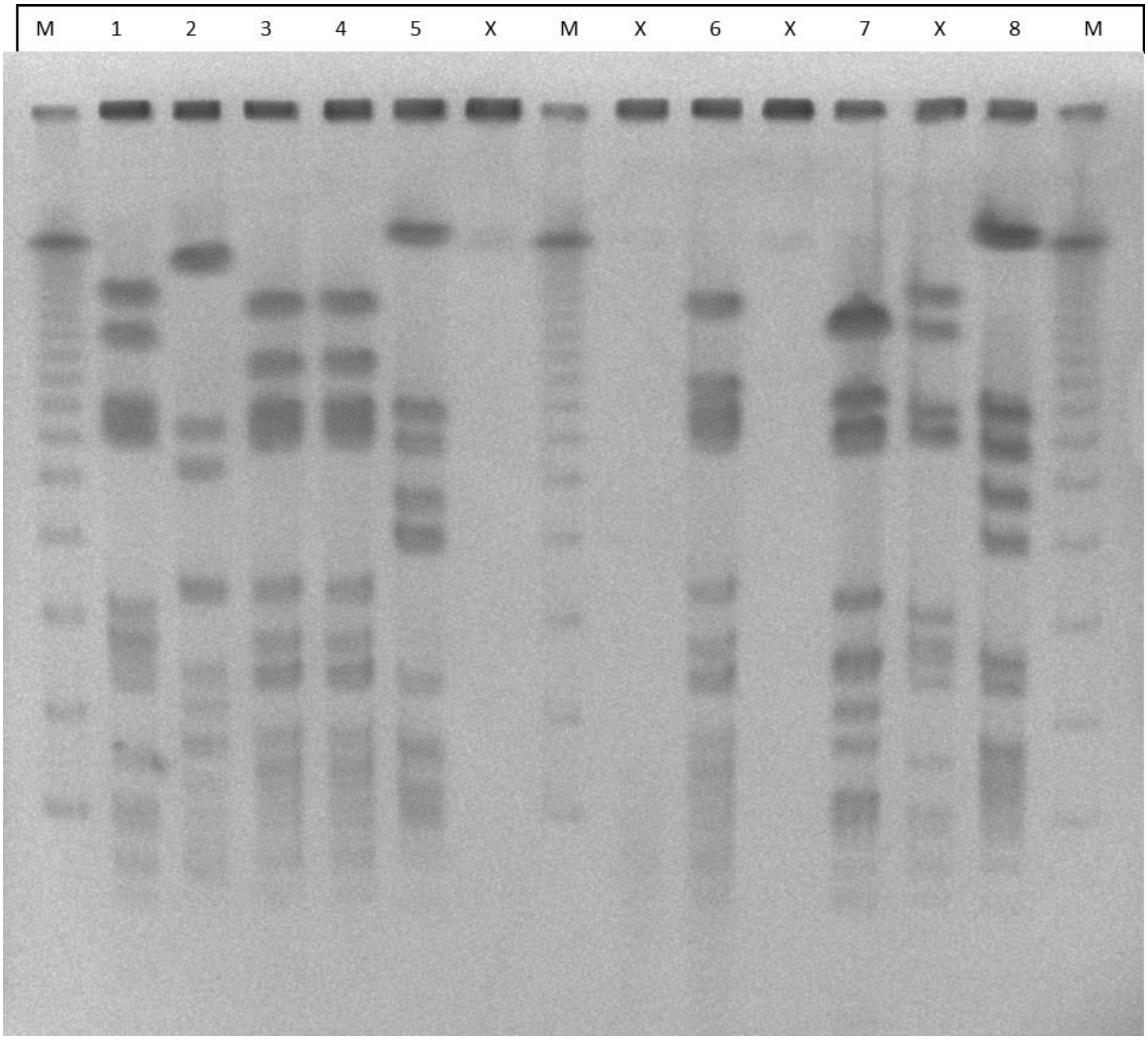

Lanes: M: molecular weight marker - lambda (λ) ladder (CHEF DNA size standard; catalog no. 170-3635; Bio-Rad) 1. BT144 2. BT151 3. BT180 4. BT192 5. BT194 6. 6/3/6 7. 6/6/15 8. PP224
