# Supplementary material for: Co-carriage of Staphylococcus aureus, Streptococcus pneumoniae, Haemophilus influenzae and Moraxella catarrhalis among three different age categories of children in Hungary
Source: PLoS One. 2020 Feb 7;15(2):e0229021. doi: 10.1371/journal.pone.0229021 (PMC7006921; doi:10.1371/journal.pone.0229021)
Supplement: S3 Raw Images — (PDF) [file pone.0229021.s006.pdf]

Fig 6 was generated from these original *S. pneumoniae* PFGE pictures.

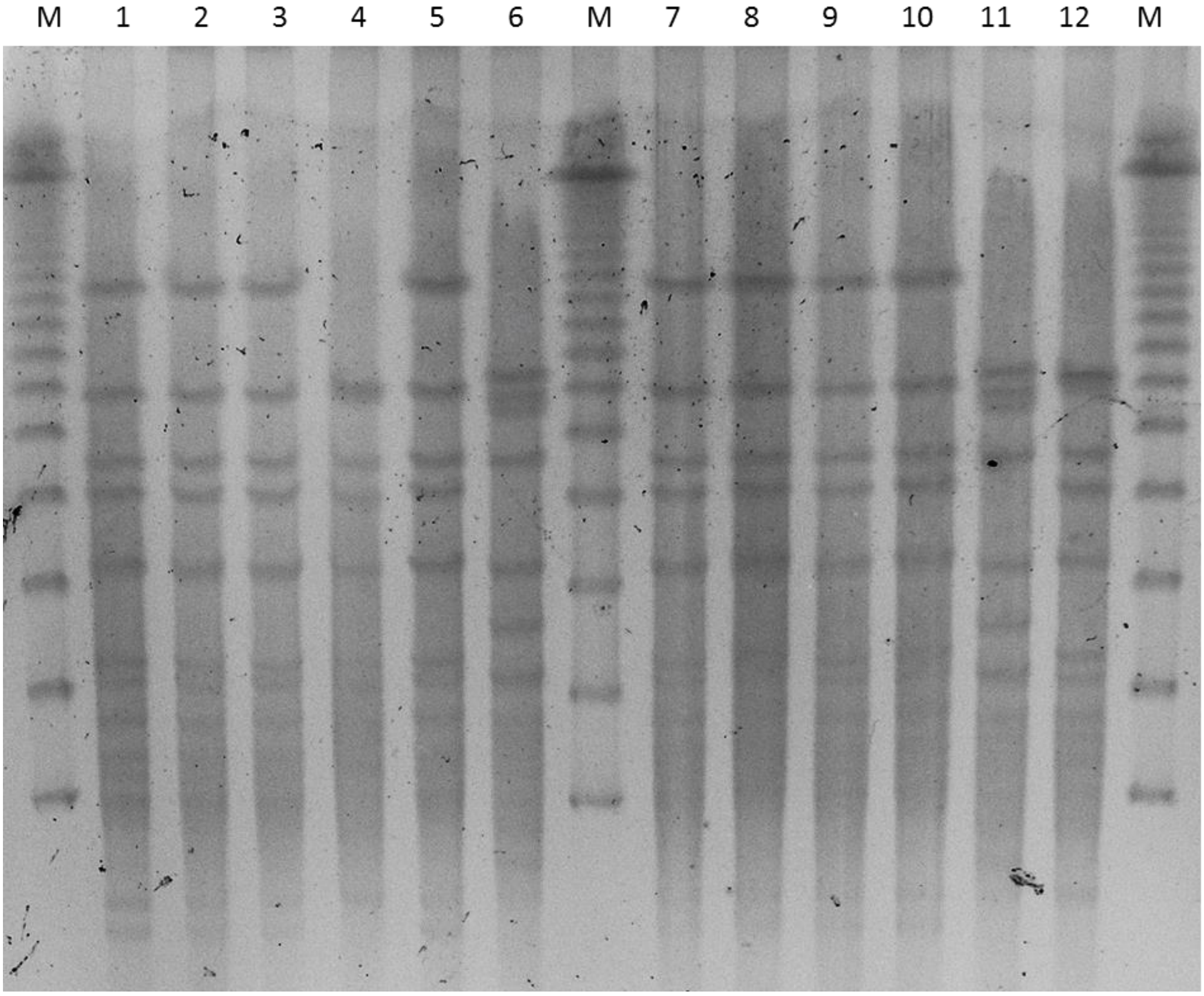

Lanes: M: molecular weight marker - lambda (λ) ladder (CHEF DNA size standard; catalog no. 170-3635; Bio-Rad) 1. PP64 2. PP83 3. PP79 4. PP38 5. PP31 6. PP76 7. PP69 8. PP68 9. PP92 10. PP65 11. PP74 12. PP39

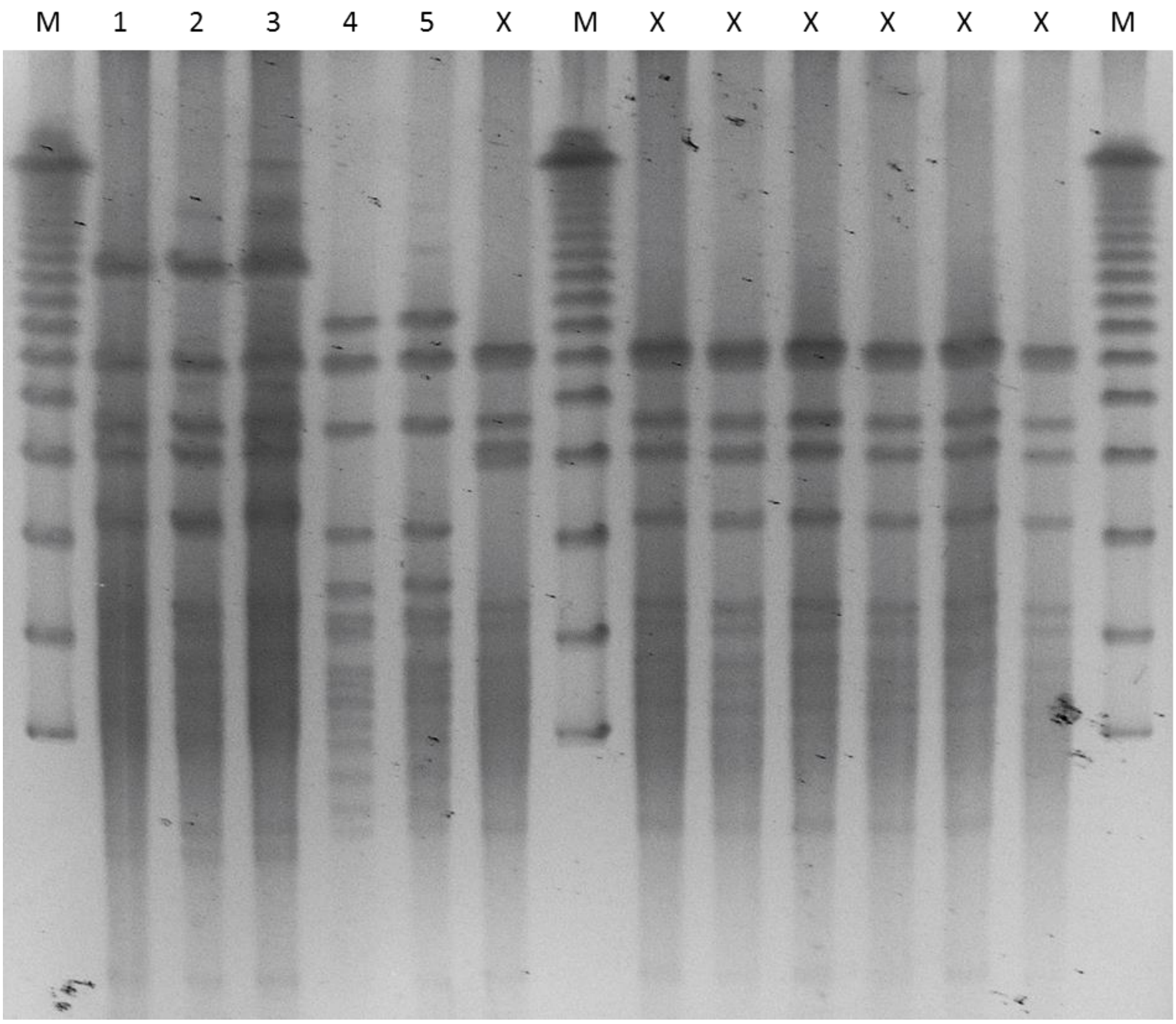

Lanes: M: molecular weight marker - lambda (λ) ladder (CHEF DNA size standard; catalog no. 170-3635; Bio-Rad) 1. PP138 2. PP178 3. PP174 4. 6/6/9 5. 6/6/10

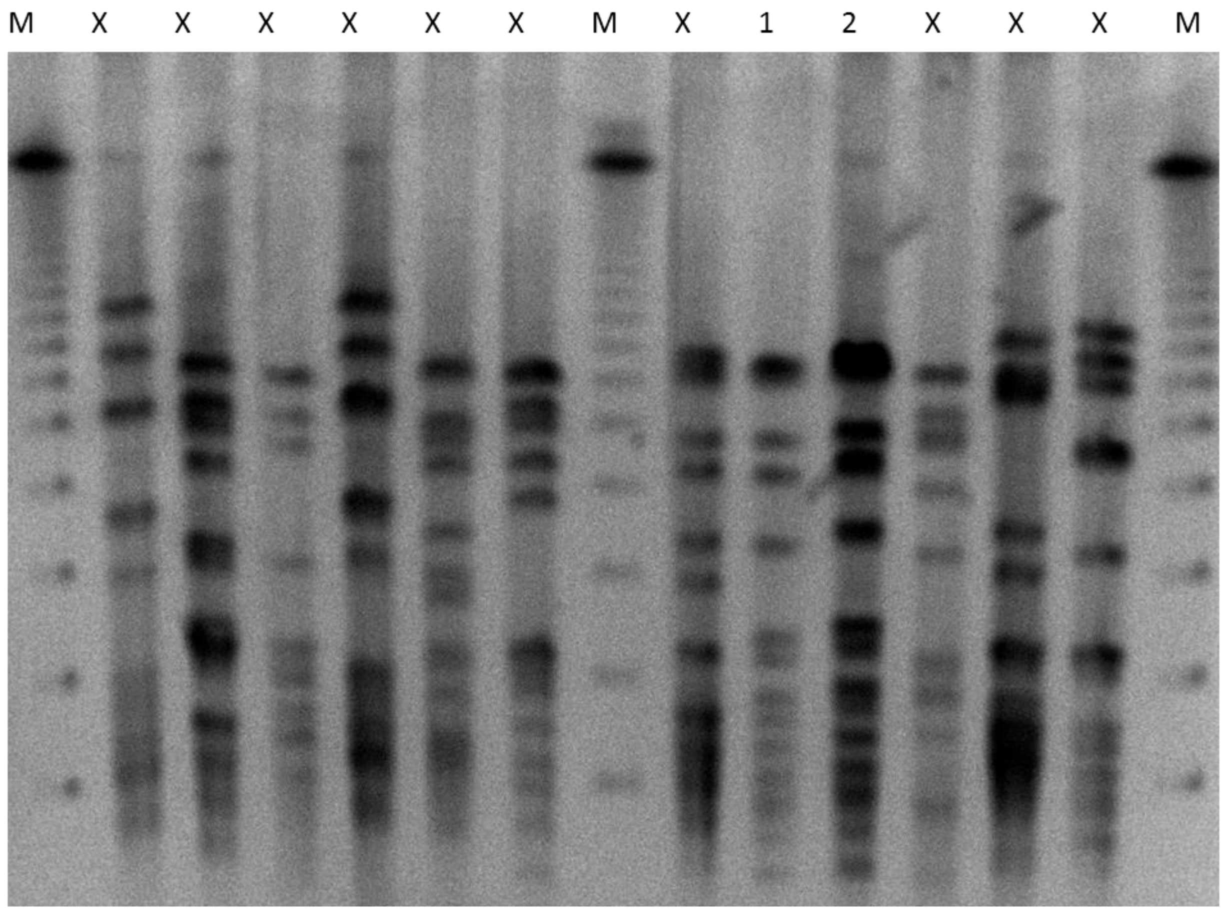

Lanes: M: molecular weight marker - lambda (λ) ladder (CHEF DNA size standard; catalog no. 170-3635; Bio-Rad) 1. BT172 2. BT71

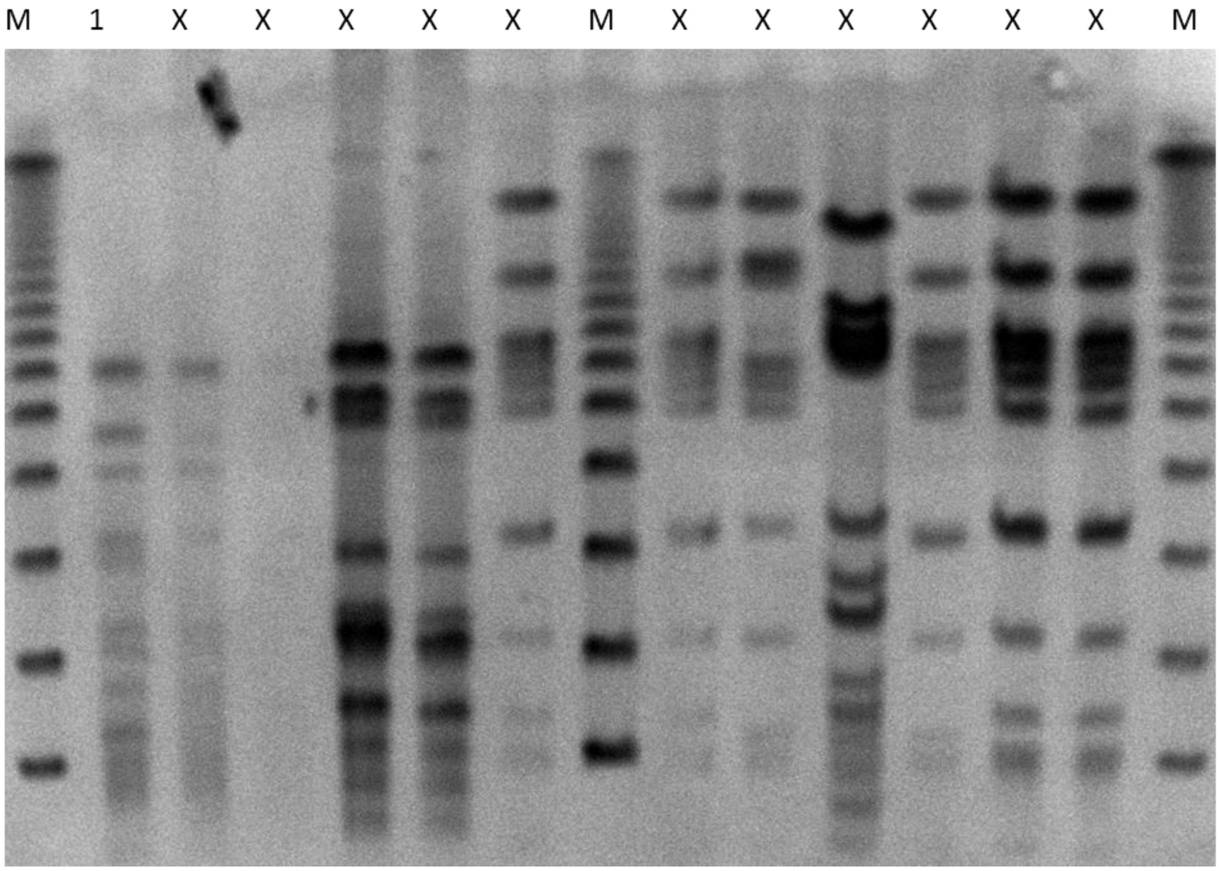

Lanes: M: molecular weight marker - lambda (λ) ladder (CHEF DNA size standard; catalog no. 170-3635; Bio-Rad) 1. BT10

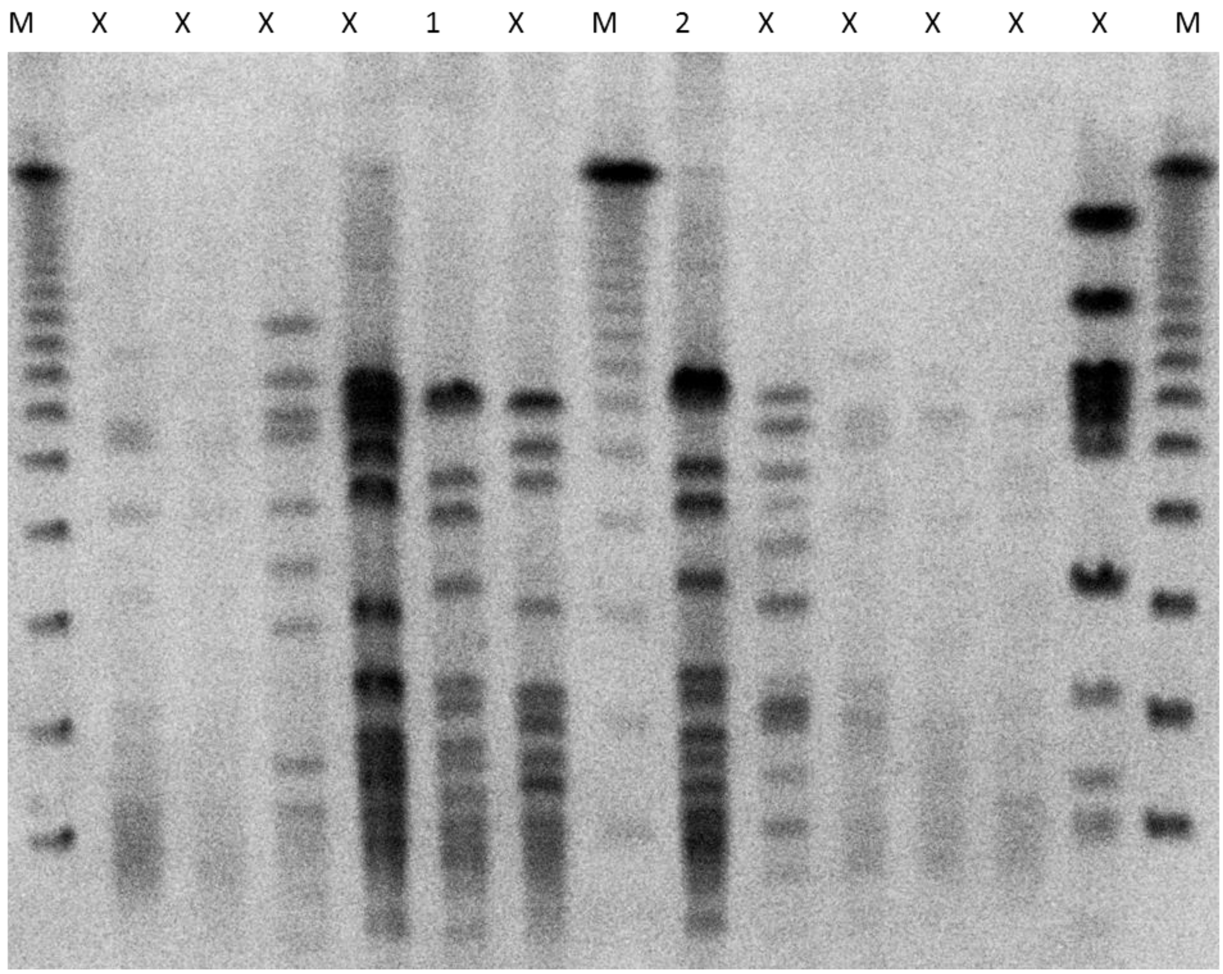

Lanes: M: molecular weight marker - lambda (λ) ladder (CHEF DNA size standard; catalog no. 170-3635; Bio-Rad) 1. BT69 2. BT196/2
